# The dorsoanterior brain of adult amphioxus shares similarities in expression profile and neuronal composition with the vertebrate telencephalon

**DOI:** 10.1186/s12915-021-01045-w

**Published:** 2021-05-21

**Authors:** Èlia Benito-Gutiérrez, Giacomo Gattoni, Manuel Stemmer, Silvia D. Rohr, Laura N. Schuhmacher, Jocelyn Tang, Aleksandra Marconi, Gáspár Jékely, Detlev Arendt

**Affiliations:** 1 Department of Zoology, University of Cambridge, Downing Street, Cambridge, CB2 3EJ UK; 2 Developmental Biology Unit, European Molecular Biology Laboratory, Meyerhofstraße 1, 69117 Heidelberg, Germany; 3 Present Address: Max-Planck Institute for Neurobiology in Martinsried, Am Klopferspitz 18, 82152 Martinsried, Germany; 4 Present Address: Department of Cell & Developmental Biology, University College London, Gower Street, London, WC1E 6BT UK; 5 Living Systems Institute, University of Exeter, Exeter, EX4 4QD UK

**Keywords:** Telencephalon, Brain evolution, Amphioxus, Neurotransmitter, Adult neurogenesis

## Abstract

**Background:**

The evolutionary origin of the telencephalon, the most anterior part of the vertebrate brain, remains obscure. Since no obvious counterpart to the telencephalon has yet been identified in invertebrate chordates, it is difficult to trace telencephalic origins. One way to identify homologous brain parts between distantly related animal groups is to focus on the combinatorial expression of conserved regionalisation genes that specify brain regions.

**Results:**

Here, we report the combined expression of conserved transcription factors known to specify the telencephalon in the vertebrates in the chordate amphioxus. Focusing on adult specimens, we detect specific co-expression of these factors in the dorsal part of the anterior brain vesicle, which we refer to as Pars anterodorsalis (PAD). As in vertebrates, expression of the transcription factors *FoxG1*, *Emx* and *Lhx2/9* overlaps that of *Pax4/6* dorsally and of *Nkx2.1* ventrally, where we also detect expression of the *Hedgehog* ligand. This specific pattern of co-expression is not observed prior to metamorphosis. Similar to the vertebrate telencephalon, the amphioxus PAD is characterised by the presence of GABAergic neurons and dorsal accumulations of glutamatergic as well as dopaminergic neurons. We also observe sustained proliferation of neuronal progenitors at the ventricular zone of the amphioxus brain vesicle, as observed in the vertebrate brain.

**Conclusions:**

Our findings suggest that the PAD in the adult amphioxus brain vesicle and the vertebrate telencephalon evolved from the same brain precursor region in ancestral chordates, which would imply homology of these structures. Our comparative data also indicate that this ancestral brain already contained GABA-, glutamatergic and dopaminergic neurons, as is characteristic for the olfactory bulb of the vertebrate telencephalon. We further speculate that the telencephalon might have evolved in vertebrates via a heterochronic shift in developmental timing.

**Supplementary Information:**

The online version contains supplementary material available at 10.1186/s12915-021-01045-w.

## Background

The telencephalon represents the most anterior part of the vertebrate forebrain. Integrating multimodal sensory input, it controls a rich repertoire of behaviours through its output circuitry [1]. Depending on the vertebrate species, it develops by eversion or evagination, always from the anterior end of the embryonic neural tube dorsal to the developing hypothalamic region [2, 3]. In all vertebrates studied, the telencephalon is initially specified by a well-known set of transcription factors. Among these, the combined regional co-expression of *foxg1* and *emx1* conveys the initial specification of the telencephalic brain region [4, 5]; *Lhx2* is required for the formation of cortical progenitors [6]; and *Pax6* and *Nkx2.1* subdivide the telencephalon into the dorsal pallial and ventral subpallial domain in mouse, zebrafish and lamprey [4, 7]. While the pallial domain gives rise to glutamatergic neurons, the subpallial domain gives rise to GABAergic neurons, of which some migrate dorsally into the pallium. The subpallial domain also gives rise to dopaminergic neurons that populate the olfactory bulb. The mature pallium thus comprises the cerebral cortex with glutamatergic and GABAergic neurons and the olfactory bulb with glutamatergic, GABA- and dopaminergic neurons. The ventral subpallium in turn comprises GABAergic neuron types constituting most of the basal ganglia [8].

The evolutionary origin of the telencephalon has been debated for centuries (see more recent contributions by [9–11]), with most authors following the ‘New Head Hypothesis’ [12] that considers the telencephalon a vertebrate innovation with no homologue in the invertebrates. While this view was mostly based on anatomical comparisons, recent comparative work across vertebrates and across chordates has shifted towards molecular comparisons with particular focus on brain regionalisation genes encoding conserved transcription factors [13–16]. The rationale is that brain parts showing similar molecular identities in comparable locations qualify as possible homologues, which can then be tested and further refined by comparisons of transmitter type and connectivity.

Following this approach, and in line with the ‘New Head Hypothesis’, no telencephalon homologue appears to be present in the tunicates, the closest relatives of the vertebrates within the chordate clade [17]. In larval ascidians, the anterior-most region of the developing neural tube does not express orthologues of the vertebrate telencephalic specification genes such as *foxg* or *emx* [16]. Instead, expression of ascidian *foxg* has been detected in the sensory palps, indicating that the telencephalon may have evolved in the vertebrate lineage via incorporation of chemosensory cells [16]—a notion supported by the double role of *foxg* in telencephalon and olfactory system specification in vertebrates [18]. Importantly, however, the ascidian larval neural tube largely degenerates during metamorphosis concomitant with the transition to sedentary lifestyle [19], indicating that the lack of expression of this particular set of transcription factors in ascidians may be due to secondary loss.

Supporting the latter, co-expression of genes orthologous to vertebrate telencephalic transcription factors has been detected in the developing brain of some non-chordate invertebrates. For example, combined expression of *foxg*, *pax6*, *lhx2* and *dach* demarcates a subregion of the brain neuroepithelium in developing annelids, from where prominent brain centres called mushroom bodies later develop [20]. Furthermore, the expression of *foxg*, *pax6*, *Lhx2* and *dach* at the base of the proboscis in one-gill-slit embryos of the enteropneust *Saccoglossus* [21–23] occurs in a region where a neural plate-like centralised component of the nervous system differentiates in adult enteropneusts [24]. These data are consistent with the notion that a brain part developing from *foxg*-, *pax6*-, *lhx2*- and *dach-*expressing neuroepithelium already existed in bilaterian ancestors (of unknown composition however, given that neuron types emerging from the annelid and enteropneust *foxg*, *Lhx2*, *Dach*, *pax6* brain regions remain to be identified). Importantly, more comparative expression and neuroanatomical data from these and other bilaterians with and without brains will be needed to substantiate this notion.

A cornerstone of any theory on telencephalic origins is the possible presence or absence of a telencephalic homologue in the invertebrate amphioxus, the most distant chordate relative of the vertebrates. Just like vertebrates and ascidians, amphioxus develops a neural tube with an anterior brain vesicle [15, 25, 26]; yet, no brain part with possible homology to the vertebrate telencephalon has so far been identified—mostly due to the lack of expression of genes orthologous to vertebrate telencephalic marker genes in the amphioxus brain vesicle at embryonic and early larval stages (see for example [15]).

To address this question, we have chosen to instead investigate the expression of these genes at later stages, namely in the post-metamorphic adult brain of the cephalochordate *Branchiostoma lanceolatum*—a life cycle stage never analysed in this context. To this aim, we serially sectioned whole amphioxus heads and reconstructed each of the patterns obtained by stitching multidimensional image datasets. We then focused on the expression analysis of genes orthologous to the major vertebrate telencephalic specification genes *FoxG1*, *Emx* and *Lhx2*; of the anterior forebrain marker *Fezf* [27]; and of dorsoventral regionalisation factors *Pax6* and *Nkx2* [28–30]. In addition, we looked at the expression of the amphioxus Hedgehog ligand, whose vertebrate counterpart plays a crucial role in telencephalic induction. In vertebrates, *Shh* is expressed in a ventromedial domain partially overlapping *Nkx2.1* [4, 31], where it activates *FoxG1* [32, 33].

We find that in stark contrast to the situation at embryonic and early larval stages, *FoxG1*, *Emx*, *Lhx2* and *Fezf* are co-expressed in the adult amphioxus brain, in a region spanning the brain vesicle from the anterior floor to the dorsalmost anterior tip (Pars anterodorsalis, PAD)—sparing only the region of the frontal eye. Across the PAD, *Nkx2.1* and *Pax6* are expressed in a complementary manner, with *Nkx2.1* covering more ventral and *Pax6* more dorsal subregions. To characterise the PAD further, we have investigated transmitter content via antibodies and the expression of synthesising enzymes or transporters. Similar to the situation in vertebrates, we detect GABA- and glutamatergic neurons, with the latter restricted to more dorsal regions, where we also locate a previously described population of dopaminergic neurons [34]. In addition, cholinergic cells populate the dorsal roof of the vesicle, where the photosensitive Joseph cells are located. Finally, we identify proliferating cells and their progeny, staining for the mitotic marker *PHH3* and via mitotic incorporation of BrdU. These data reveal patterns of ventricular growth with differentiating neurons located more basally in a thickened, pseudostratified epithelium.

Our data suggest that part of the adult brain in ancestral chordates may have been specified by *FoxG1*, *Emx* and *Lhx2* and regionalised by *Nkx2.1* and *Pax6*. This brain region would have also contained different populations of GABAergic neurons, and a dorsal portion with GABA-, dopamine- and glutamatergic neurons that resembled today’s olfactory bulb in terms of neuron type composition. This ‘pre-telencephalic rudiment’ would have subsequently given rise to the telencephalon in vertebrates and PAD in amphioxus.

## Results

### Serial sectioning to study adult amphioxus brain anatomy

**Fig. 1:**
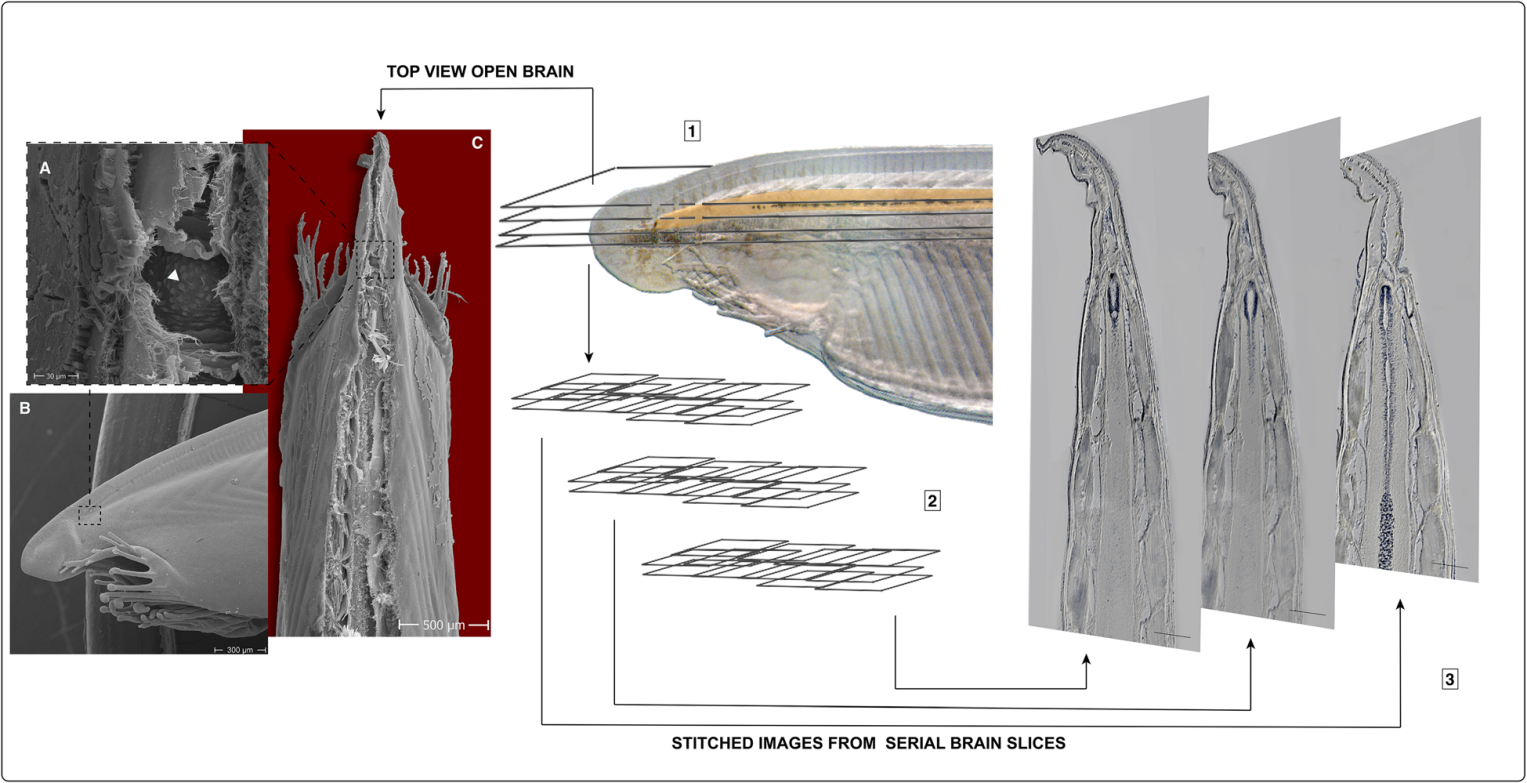
Whole-brain serial sectioning and image reconstruction pipeline. **a** Scanning electron microscopy (SEM) image showing a magnified view of the open brain in **c**, revealing big Joseph cells on the top layer (arrowhead). **b** SEM image showing a lateral view of an amphioxus head. **c** SEM image showing a dorsal image of an amphioxus head opened dorsally at the level of the brain and neural tube. Pipeline is shown as steps: (**1**) coronal sections taken at the level of the brain, (**2**) multidimensional imaging of hybridised sections, and (**3**) stitching of multiple images to reconstruct patterns across the entire brain. Scale bars in 3 are 125 μm, otherwise as indicated

To study anatomy and gene expression in the amphioxus brain, we used a similar method to that described for serial vibratome sectioning of adult amphioxus heads [35]. Either paraffin or vibratome sections were obtained after tissue fixation and cut to a thickness of 12–14 μm or 60 μm, respectively. These were stained either in slides or in floating sections and imaged using automatic multidimensional pipelines (Leica LAS X or Zeiss Zen) for large-area tiles. Tiles were subsequently stitched to obtain cellular-resolution images of the entire amphioxus head. The series of sections (between 10 and 12 sections per head) covered the entire volume of the brain vesicle and parts of the neural tube (up to the level of the 6th somite). This pipeline allowed us to analyse gene expression patterns and immunostainings for the entire anterior brain at cellular resolution (see Fig. 1 and Additional files 1, 2, 3, 4, 5, 6, 7, 8, 9, and 10 Figures S1-S10).

### Transcription factors involved in telencephalon development are expressed in the adult amphioxus brain

**Fig. 2:**
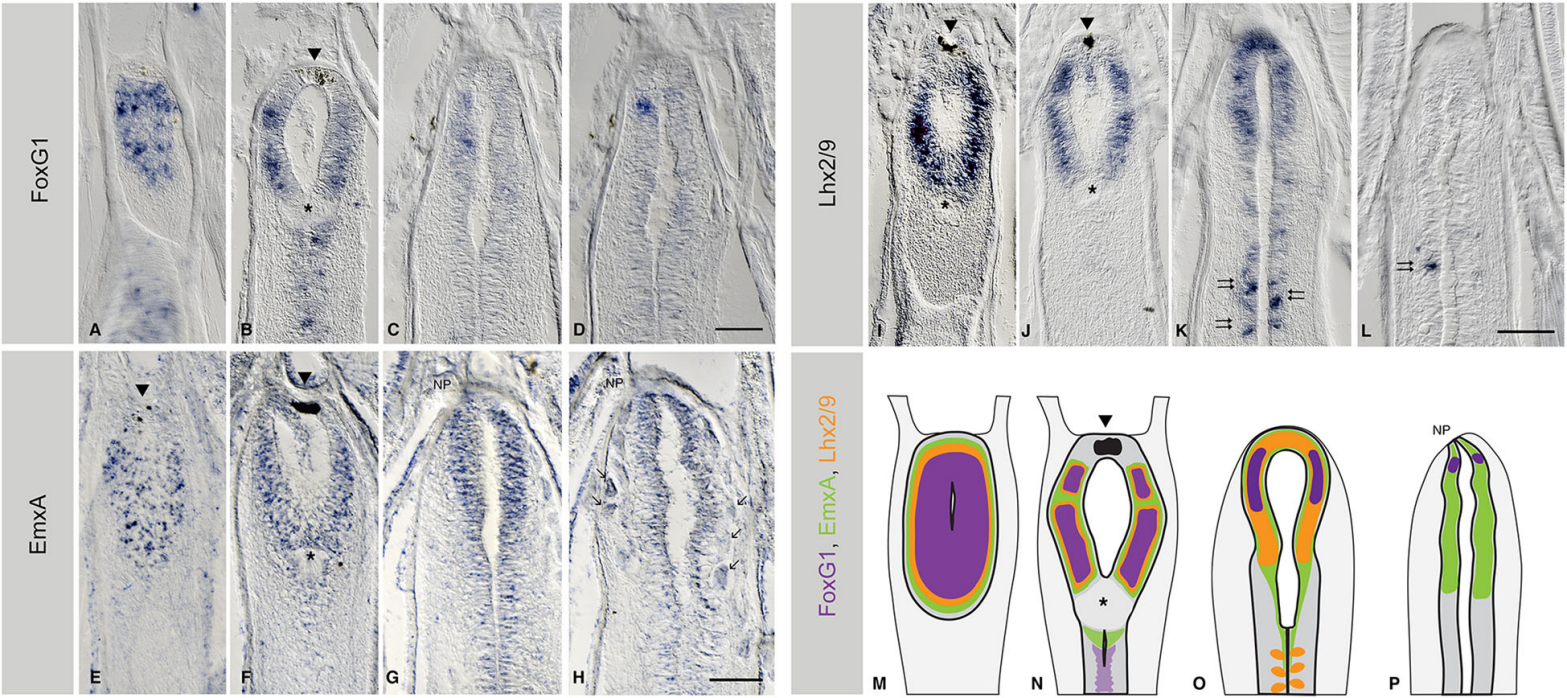
Expression of telencephalic genes in the adult brain of amphioxus. **a**–**d** The expression of *Bf1/FoxG1*, **e**–**h** *EmxA*, and **i**–**l** *Lhx2/9*, all shown in serial coronal paraffin sections from ventral to dorsal, with the ventricle of the cerebral vesicle (cv) centred in the images. In all cases, the anterior part of the brain is at the top of the image. *FoxG1* expression extends throughout the ventral and lateral walls of the cv in adults (**a**–**d**). *EmxA* (**e**–**h**) is expressed in the dorsal three-quarters of the cerebral vesicle. Only *EmxA* reaches the top mantle of Joseph cells, indicated with arrows in **g** and **h**. *Lhx2/9* expression (**i**–**l**) spans throughout the dorsal half of the cv with a dorsal limit just below the mantle of Joseph cells. **m**–**p** summarises the expression of all the genes at the respective sections. All scale bars in amphioxus sections are 50μm. Arrowheads indicate the pigment of the frontal eye. Asterisks indicate the position of the infundibular organ. Arrows indicate the position of the Joseph cells. Double arrows indicate the pattern that is similar in Lhx2/9 to the summed expression of orthologous genes in the zebrafish hindbrain and reticulo-spinal neurons. Abbreviation: NP neuropore. Full series of sections are shown in Additional files 1, 2 and 4 Figures S1-S2 and S4

**Fig. 3:**
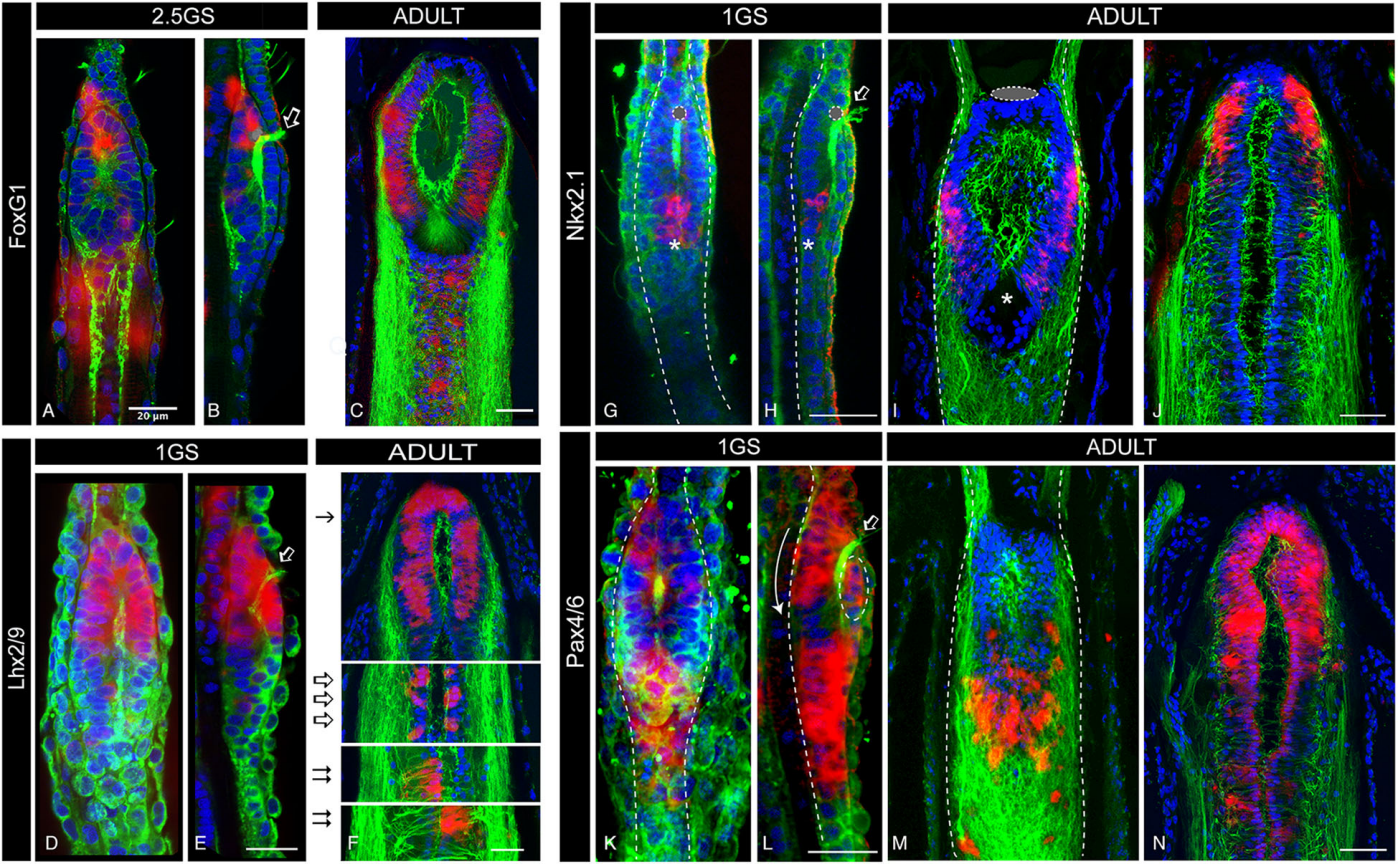
Post-metamorphic expansion of domains expressing telencephalic genes in amphioxus. **a**–**c** Confocal images of FoxG1 expression in 2.5GS embryos (**a** dorsal and **b** lateral view) and adult (**c**) brains, showing the great expansion of the FoxG1 domain in adults. In embryos, only few cells under the fontal eye are visible (**b**). **c** A confocal image of the paraffin section shown in Fig. 2b. **d**–**f** Confocal images of Lhx2/9 expression in a 1GS embryo (**d** dorsal and **e** lateral view) and an adult (**f**) brain. In embryos, Lhx2/9 is expressed only in the anterior half of the brain (**d**, **e**), while in adults (**f**) it occupies the entire cerebral vesicle and expands posteriorly into paired clusters of cells, similar to those observed in zebrafish at the midbrain/hindbrain boundary (empty arrows in **f**) and in the reticulo-spinal system (double arrows in **f**). **f** A confocal image of the paraffin section shown in Fig. 2k and l. **g**–**j** Confocal images of Nkx2.1 expression 1GS embryos (**g**, **h**) and adult (**i**, **j**) brains, showing that in early stages of development only a few Nkx2.1+ cells are located in the ventral-caudal side of the brain, close to the infundibular organ (*in **g** and **h**). This contrast with the anterior expansion in adults on the ventral side of the brain (**i**) and a new dorsal-anterior site of Nkx2.1 expression (**j**), previously undetectable in larval brains. **i**, **j** Confocal images of paraffin sections in Additional file 6 Figure S6. **k**–**n** Confocal images of Pax4/6 expression in 1GS embryos (**k**, **l**) and adult (**m**, **n**) brains, showing that the dorsal-anterior domain is greatly expanded in adults (**n**) while in the larvae is only composed of a few cells (dashed circle in **l**). **m**, **n** Confocal images of paraffin sections in Additional file 5 Figure S5. Empty arrow in **b**, **e**, **h** and **l** indicates cilia projecting from within the brain through the neuropore, highlighting the brain ventricle that divides the brain into a dorsal and a ventral side in embryos. Colour code: red, gene expression; green, neuropil (acetylated tubulin); blue, nuclear staining (DAPI). All scale bars in amphioxus adult sections are 50μm, while in embryos are 20μm. Abbreviations: 1GS one-gill-slit stage, 2.5GS 2.5 gill-slit stage

Taking advantage of our pipeline, we investigated the expression of *FoxG1*, *Emx*, *Lhx2*, *Fezf*, *Nkx2.1* and *Pax4/6* transcription factors in the amphioxus post-metamorphic cerebral vesicle (see Fig. 2 and Additional file 10 Figure S10). We found *FoxG1* expressed in a broad anterior domain that occupies a large part of the cerebral vesicle (see Fig. 2a–d, Additional file 1 Figure S1 and Additional file 10 Figure S10), extending from the floor of the cerebral vesicle (Fig. 2a) to a point about 14–16 microns above, before the ventricle closes dorsally by the Joseph cells, a specialised cell type that caps the brain of adult amphioxus (Fig. 1a). This contrasts with the limited expression of *FoxG1* earlier in development, when it is expressed in a small number of ventral cells located under the pigment of the frontal eye (Fig. 3a–c), as previously described [36]. The broader expression of *FoxG1* in adult amphioxus brains is similar in pattern to the earliest expression of *FoxG1* in E8.5 mouse embryos before the telencephalon subdivides into paired vesicles [37]. At this stage of development, the mouse telencephalon shows no morphological subdivisions [4, 38]. The adult amphioxus brain resembles this, in that it contains few morphological landmarks other than the anterior pigment of the frontal eye and the more posterior infundibular organ (IO), a secretory organ situated at the caudal end of the cerebral vesicle from which the Reissner’s fibres emanate [39]. The expression of *FoxG1* in adult amphioxus is primarily contained between these two landmarks (Fig. 2b). In vertebrates, the *FoxG1* domain later segregates as the brain sub-regionalises, a re-modelling driven by both proliferation and apoptosis [31, 40–42].

In mice, the dorsal portion of the *FoxG1* domain partially overlaps with the expression of *Emx1* [28]. We set out to explore if this was also the case in amphioxus by examining the expression of two of the three *Emx* genes present in the amphioxus genome: *EmxA* and *EmxB* [43, 44]. The three amphioxus *Emx* genes (*EmxA*, *EmxB* and *EmxC*) are amphioxus-specific duplicates of the pro-orthologous *Emx* gene, which gave rise to the vertebrate *Emx1* and *Emx2*. Accordingly, they are equally related to both *Emx1* and *Emx2* [44, 45]. Amphioxus *Emx* genes have been characterised at the sequence level but no expression has so far been reported. We observed that both *EmxA* and *EmxB* were broadly expressed in the adult cerebral vesicle, with a ventral limit in the upper ventricle floor, a rostral limit behind the frontal eye and a caudal limit coincident with the IO (Fig. 2e–h, Additional file 2 Figure S2 and Additional file 3 Figure S3). *EmxB* shows a more restricted expression mostly overlapping the expression of *EmxA* (Fig. 2e–h and Additional file 10 Figure S10), with the exception of the most dorsal side, where only *EmxA* is weakly expressed by the Joseph cells (see arrows in Fig. 2g–h).

In vertebrates, *Lhx2* and *Lhx9* are also essential for telencephalon development [46, 47]. Consistent with the expression of *FoxG1* and *EmxA/B*, the single amphioxus *Lhx2/9* orthologue is also widely expressed in the cerebral vesicle of the adult amphioxus, yet absent from the vesicle floor (Fig. 2i–l and Additional file 4 Figure S4). As in the vertebrate telencephalon, the anterior *Lhx2/9* domain overlaps with the expression of *FoxG1* and *Emx* in the anterior cerebral vesicle (Fig. 2m–p). Moreover, the patch-like pattern of *Lhx2/9* in the posterior amphioxus brain (beyond the IO) is consistent with similar domains in the zebrafish embryo, where *Lhx2* and *Lhx9* also mark the midbrain/hindbrain boundary and the bilaterally arranged reticulo-spinal neurons [46] (Fig. 2k–l (double arrows), Fig. 2o and Fig. 3f (empty arrows and double arrows)). In amphioxus, the most posterior bilaterally arranged *Lhx2/9*+ neurons are asymmetrically paired (Fig. 2o, Fig. 3f double arrows and Additional file 4 Figure S4), probably following the left-right offset of the somites and associated nerve roots. For comparison, we also analysed the embryonic expression of *Lhx2/9*, finding it confined to the most anterior part of the cerebral vesicle, with a caudal limit roughly at the middle of the ventricle in one-gill-slit embryos (Fig. 3d–e).

### The adult amphioxus brain shows dorsoventral regionalisation

**Fig. 4:**
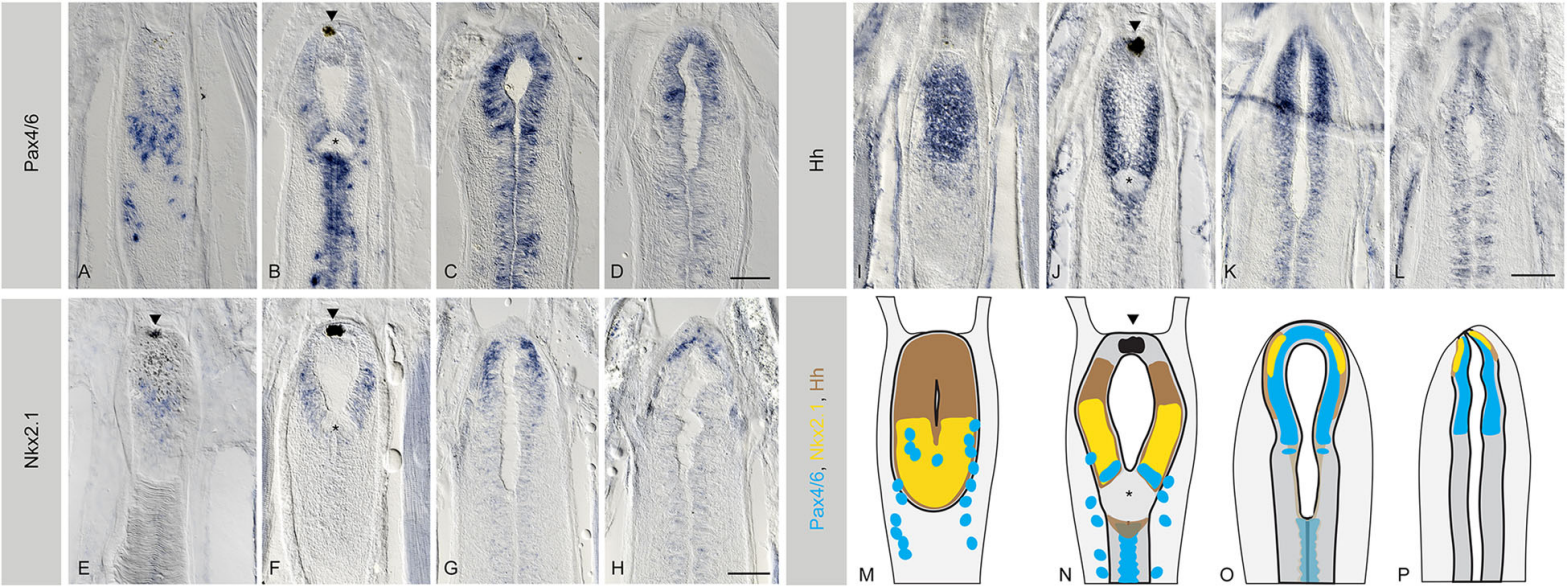
Late regionalisation of the telencephalic-like domain in amphioxus. **a**–**d** The expression of *Pax4/6*, **e**–**h** *Nkx2.1* and **i**–**l** *Hh*, all shown in serial coronal paraffin sections from ventral to dorsal, with the ventricle of the cerebral vesicle (cv) centred in the images. In all cases, the anterior part of the brain is at the top of the image. **a**–**d** Pax4/6 is expressed ventrally in the posterior half of the cerebral vesicle but in a salt-and-pepper pattern (**a**, **b**) and progressively anteriorises as the expression becomes dorsal (**c**, **d**). **e**–**h** Nkx2.1 is also expressed ventrally in the posterior half of the cerebral vesicle (**e**–**f**), but as in the case of Pax4/6, it becomes anterior in the upper layers of the brain, where it marks the anterior neural border (**g**, **h**). **i**–**l** Hh, on the other hand, is broadly expressed throughout the cv, with expression gradually fading away in a dorsal direction (this is best appreciated in Additional file 7 Figure S7). **m**–**p** summarises the expression of all the genes at the respective sections. All scale bars in amphioxus sections are 50μm. Arrowheads indicate the pigment of the frontal eye. Asterisks indicate the position of the infundibular organ. Abbreviation: NP neuropore. Full series of sections are shown in Additional files 5, 6 and 7 Figures S5-S7

We next explored the expression patterns of genes with known roles in dorsoventral regionalisation within the vertebrate telencephalon. In amphioxus, *Pax4/6*, the orthologue of vertebrate *Pax6*, has been studied in early embryos, where it is expressed in some cells of the frontal eye [48–50], in some cells of the ventral cerebral vesicle and in the lamellar body, a putative photoreceptive structure located in the dorsal roof of the cerebral vesicle (Fig. 3k, l). We observed a very different pattern in the adult cerebral vesicle, with the ventral-anterior portion of the brain showing no expression of *Pax4/6* (Fig. 3k–n, Fig. 4a–d and Additional file 5 Figure S5) and the dorsal half showing expanded expression. This dorsal *Pax4/6* domain is no longer associated with the lamellar body, which in adult brains is fragmented into scattered lamellar cells [26], but overlaps with the *Emx* and *Lhx2/9* domains, as observed in the developing vertebrate telencephalon [30]. In this overlapping region, *Pax4/6* expression reaches the anterior neural border, in a domain located just above the neuropore (Fig. 4c, d, Additional file 5 Figure S5 and Additional file 10 Figure S10). This is comparable to the *Pax6* domain in the vertebrate telencephalon, which is also positioned at the most anterior-dorsal side of the brain. Contrasting *Pax4/6* expression in one-gill-slit embryos and in adults, we noted that in adults the ventral domain is posteriorly shifted towards the mid-caudal end of the cerebral vesicle (Fig. 3m). There, it extends caudally around the IO and along the central canal, where a small number of *Pax4/6* expressing cells had already been observed in larvae [50].

In the developing vertebrate forebrain, *Nkx2.1* expression in the ventral telencephalon depends on *FoxG1*, whereas *Nkx2.1* hypothalamic expression does not [40, 41]. In adult amphioxus brains, we also observe two interconnected subdomains of *Nkx2.1* expression. First, *Nkx2.1* is expressed in the posterior half of the ventral vesicle, with a posterior limit very close to the IO (Fig. 4e, f and Additional file 6 Figure S6). This ventral posterior expression is consistent with that previously described in seven-somites neurulating embryos [15], and in one-gill-slit embryos (Fig. 3g, h). As only a few anterior cells express *FoxG1* at embryonic stages (Fig. 3a, b), this suggests that *FoxG1* might not be required to initiate the expression of *Nkx2.1* in this ventral domain. However, our data demonstrate that *Nkx2.1* expression in adults extends towards the anterior part of the brain, reaching the tip of the vesicle more dorsally above the pigment spot of the frontal eye (Fig. 4g, h). There, it overlaps with the *FoxG1* domain at least partially (see Fig. 2 and Additional file 1 Figure S1 for comparison), which would allow for interactions with *FoxG1*, just like Nkx2.1 and FoxG1 interact in the vertebrate telencephalon.

Expression of *Hh* has previously been reported in the neural plate and underlying notochord of neurulating amphioxus embryos. However, no expression has been detected in the cerebral vesicle or anterior neural plate at embryonic stages [51, 52]. In vertebrates, the expression of *Shh* in the anterior neural plate is required to induce the formation of the telencephalon [32]. Consequently, the absence of *Hh* expression in the amphioxus cerebral vesicle during embryogenesis has been interpreted as reflecting the absence of a telencephalon. In contrast, we found that in the adult amphioxus brain, *Hh* is strongly expressed in the cerebral vesicle, largely overlapping expression of *FoxG1* (Fig. 4i–l and Additional file 10 Figure S10) and *Nkx2.1* (Fig. 4e–h Additional file 10 Figure S10). In vertebrates, *Shh* and *FoxG1* also overlap and conditional removal of hedgehog signalling using a *FoxG1* driver results in a loss of ventral telencephalon patterning [33]. Furthermore, *Shh* and *Nkx2.1* co-expression is a conserved characteristic feature of the vertebrate MGE (Medial Ganglionic Eminence), as well as of the recently discovered MGE in cyclostomes [31]. Moreover, the MGE appears to be a major source of cortical neurons in all vertebrates [53–55].

### The amphioxus brain grows progressively and outwards from the ventricular surface

**Fig. 5:**
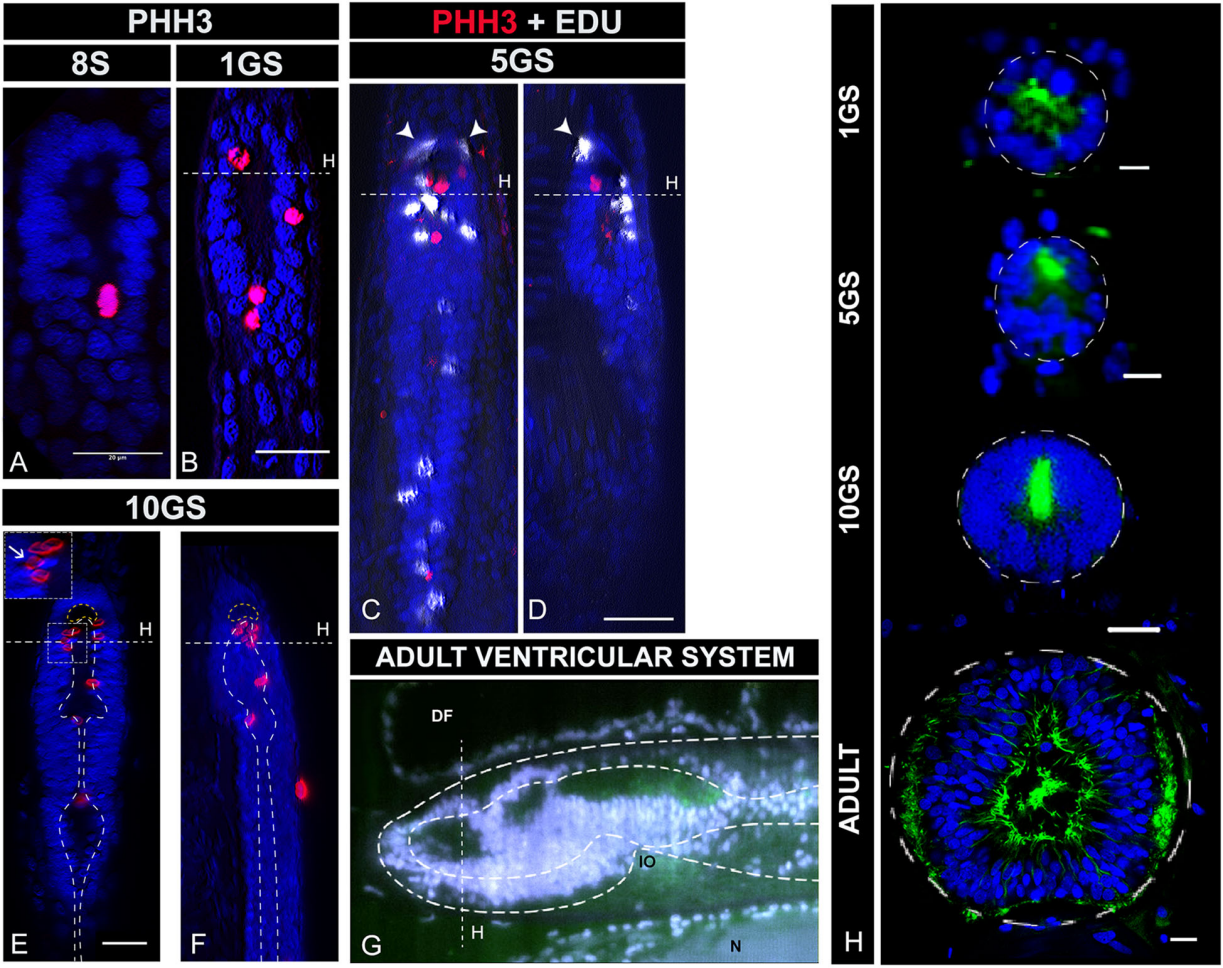
Late growth of the amphioxus brain. **a**–**f** Proliferation patterns in the brain throughout early and late development assessed by PHH3 (red) immunohistochemistry in whole mount preparations. PHH3 is first detected in the brain of amphioxus at the mid-neurula stage (8 somites: 8S) (**a**: dorsal view). Thereafter, PHH3 persists in the ventricular zone of the growing brain as shown in 1GS embryos (**b**: dorsal view), in 3-month-old embryos (5GS) (**c**: dorsal view and **d**: lateral view) and in pre-metamorphic embryos (10GS) (**e**: dorsal view and **f**: lateral view). At all stages, mitotic cells (PHH3+) are localised in the ventricular surface (outlined in **e** and **f**) and only post-mitotic cells (Edu: white labelling in **c** and **d**) are pushed outwards away from the ventricle. Arrowheads in **c** and **d** indicate Edu+ cells that divided in the ventricle and migrated basally. Occasionally, it is possible to observe the apical-basal translocation of daughter cells, as indicated by an arrow in the inset in **e**. **g** Lateral view of the ventricular system of the adult brain showing the presence of at least three ventricles. The image is taken from a whole brain stained and clarified using confocal microscopy. Staining in grey shows the nuclei of the brain cells. Staining for acetylated tubulin in green reveals ventral neuropil and the ciliated lumen of the ventricles. **h** Frontal sections of amphioxus brains at the levels indicated in the rest of the panels, showing the growth and development of the ventricular system. The dorsal side of the brain begins thickening at pre-metamorphic stages (10GS) and continues to grow to show a very different morphology in adults. Colour code: blue, brain nuclei; green, neuropil and intraventricular cilia (acetylated tubulin). All scale bars are 20μm, except in **h** that are 10 μm. Abbreviations: 8S 8 somites neurula, 1GS one-gill-slit embryo, 5GS five-gill-slit larva (3 months old), 9GS nine-gill-slit larva (pre-metamorphic), 10GS ten-gill-slit larva (pre-metamorphic), DF dorsal fin, IO infundibular organ, N notochord

Our comparative expression analysis between embryonic and adult brains (Fig. 3) indicates that territories of new genetic identity arise throughout the life cycle and after metamorphosis. To understand how the continued development and differentiation of the amphioxus vesicle occurs, we analysed brain proliferation patterns from early neurulation (formation of the neural tube) to metamorphosis. We profiled developing brains at single cell resolution using the mitotic marker *PHH3*, whose phosphorylation dynamics in amphioxus were not previously known. The earliest *PHH3+* cells occur rather late in the embryonic brain, not at the initial phases of brain formation but in embryos with seven to eight somites (Fig. 5a). Although surprising, this is supported by previously published BrdU-pulse experiments in developing amphioxus, which show a burst of BrdU incorporation (cells in S-phase) in the brain of embryos at mid-neurula stages but not earlier [56]. From this stage onwards, *PHH3*+ cells remain sparsely distributed over the ventricular surface of the brain (Fig. 5a–f), indicating that neural stem cells divide apically in the ventricular zone of the neuroepithelium, as in vertebrates [57]. The number of *PHH3*+ cells increases as the brain grows, yet it remains low in comparison to vertebrates. We hypothesise that this explains why in amphioxus the brain tissue does not bulge out as in vertebrates. Notably, while early *PHH3*+ cells are mostly located on the ventral side of the brain, they are more numerous in the dorsal and anterior brain at later stages (Fig. 5f).

By pulse-labelling dividing cells with Edu (5-ethynyl-2′-deoxyuridine) at the five-gill-slit stage (Fig. 5c, d), we further found that cells seem to divide in the ventricle and, following mitosis, they translocate basally to outer layers of the brain (see arrowheads in Fig. 5c, d and mitotic nuclei pushed away from the ventricular surface indicated with an arrow in Fig. 5e). This is similar to the reported behaviour of progenitor cells in the ventricular zone of the mouse telencephalon [57]. This anterior inside-out growth (from the ventricle) over the lengthy period of late development of amphioxus is consistent with, and could thus explain, the posteriorisation of the *Nkx2.1* and *Pax6* domains in the ventral cerebral vesicle and the development of an anterior new tissue, with additional cells expressing *FoxG1* at later stages.

Cross-sectional analysis of the amphioxus brain at these developmental stages is consistent with our proliferation assays, showing that the dorsal-ventral relative thickness of the brain changes over developmental time (Fig. 5h). At the mid-neurula stage, for example, the brain is still open with no brain roof. The tubular shape of the brain only begins to be noticeable around the one-gill-slit stage, when the brain is formed by a single layer of cells all around the ventricle (Fig. 5h 1GS). In the subsequent 3 months of development, the brain thickens in the ventral side (Fig. 5h 5GS). It is only around metamorphosis that considerable thickening also occurs in the dorsal side (Fig. 5j 10GS). This late growth agrees with the complex ventricular system that we observed in adults, with a marked internal regionalisation that probably arises as a consequence of this differential growth (Fig. 5g and 5h-ADULT).

### Late neuronal differentiation in the amphioxus brain generates dorsoventrally restricted populations of glutamate-, GABA-, dopamine-, and cholinergic neurons

**Fig. 6:**
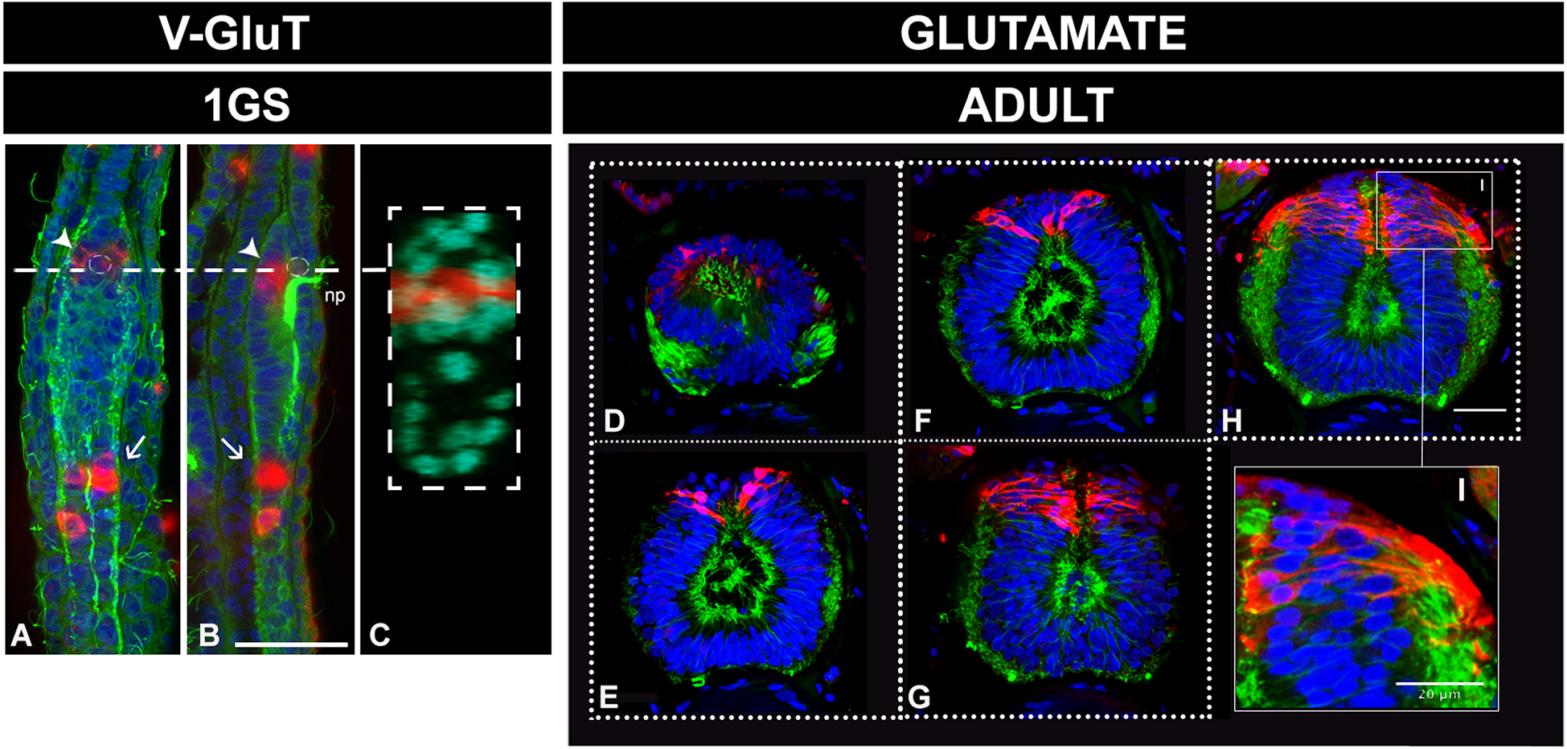
Pallial-like accumulation of glutamatergic neurons in the adult amphioxus brain. **a**–**c** Distribution of glutamatergic neurons in one-gill-slit embryos (1GS) detected by in situ hybridisation of *VGluT* in whole mount preparations. Glutamatergic neurons at these stages are mostly located in the neural tube (arrows), with the exception of a couple of cells located beneath the pigment of the frontal eye, as indicated by the arrowheads. Their ventral position is obvious in a cross section of the same embryo (**c**) at the level of the dashed line in **a** and **b**. **d**–**i** Distribution of glutamatergic neurons in the adult amphioxus brain, detected by immunohistochemistry and shown in frontal thick vibratome sections ordered in an anterior-to-posterior direction. Glutamatergic neurons are dorsally restricted in the anterior part of the cv. Neurons in this area can also be observed in the coronal sections shown in Additional file 9 Figure S9. **i** A magnified view from panel **h** showing the pseudostratified structure of the neuroepithelium. All scale bars are 20μm. Colour code: red, VGluT/glutamate; green, neuropil and intraventricular cilia (acetylated tubulin); blue/cyan, nuclear staining (Dapi). Abbreviation: np neuropore

**Fig. 7:**
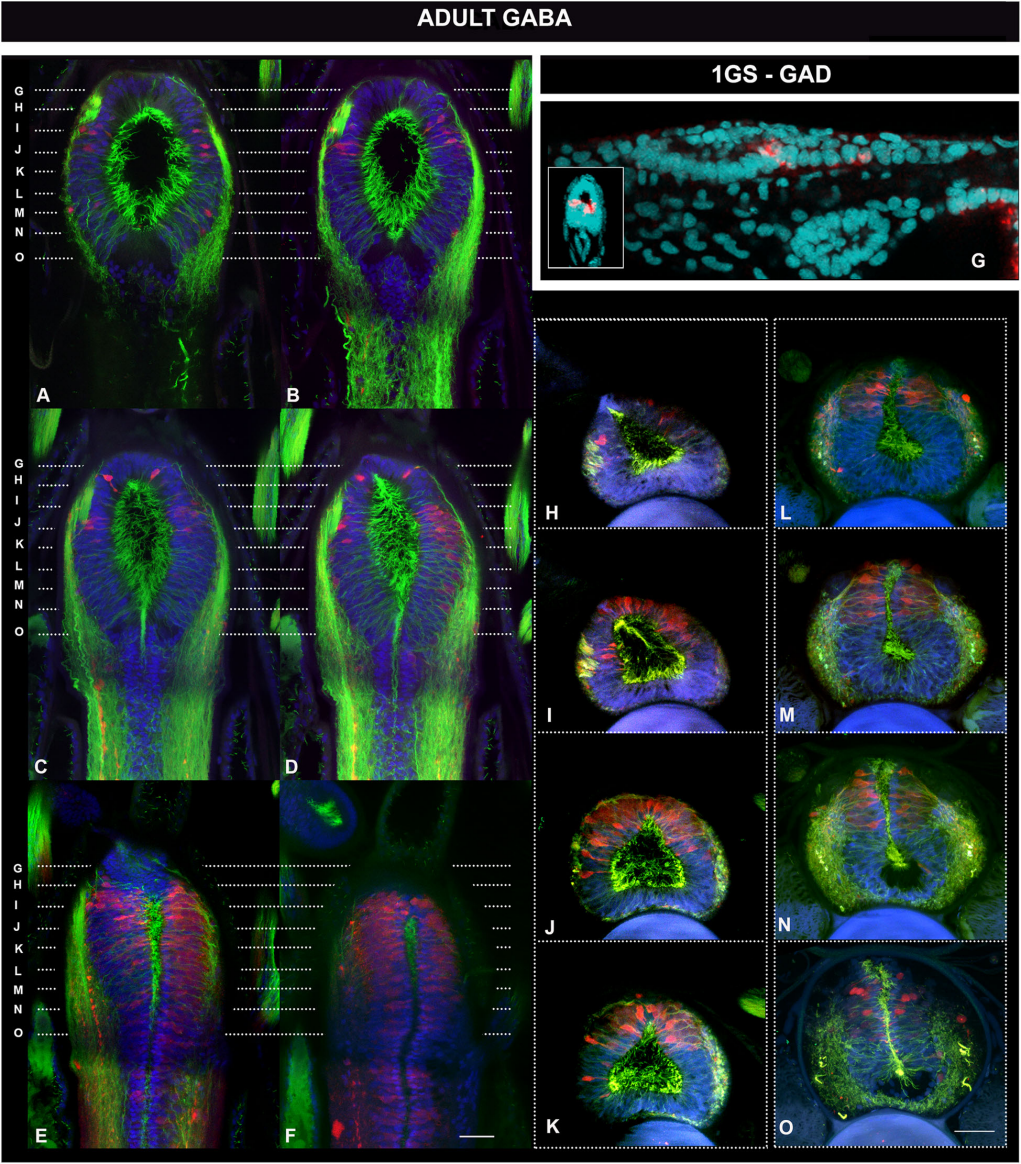
Pallial-like accumulation of GABAergic neurons in the adult amphioxus brain. **a**–**f**, **h**–**o** Distribution of GABAergic neurons in the adult amphioxus brain, detected by immunohistochemistry and shown in coronal (**a**–**f**) and frontal (**h**–**o**) thick vibratome sections. Planes of sectioning for frontal sections are indicated by dashed lines on the coronal sections. Coronal sections are ordered from ventral to dorsal (**a** to **f**) and frontal sections are ordered from anterior to posterior, as indicated by the dashed lines in **a**–**f**. GABAergic neurons concentrate in the dorsal half of the cv, in this region of the central nervous system. **g** Distribution of GABAergic neurons in one-gill-slit embryos (1GS) detected by in situ hybridisation of GAD in whole mount preparations. GABAergic neurons are at these stages located in the neural tube. Only a pair is visible at the caudal end of the cv, as indicated by the dashed line. Their ventral position is obvious in a cross section of the same embryo, shown in the inset. All scale bars are 20μm. Colour code: red, GAD/GABA; green, neuropil and intraventricular cilia (acetylated tubulin); blue/cyan, nuclear staining (Dapi)

**Fig. 8:**
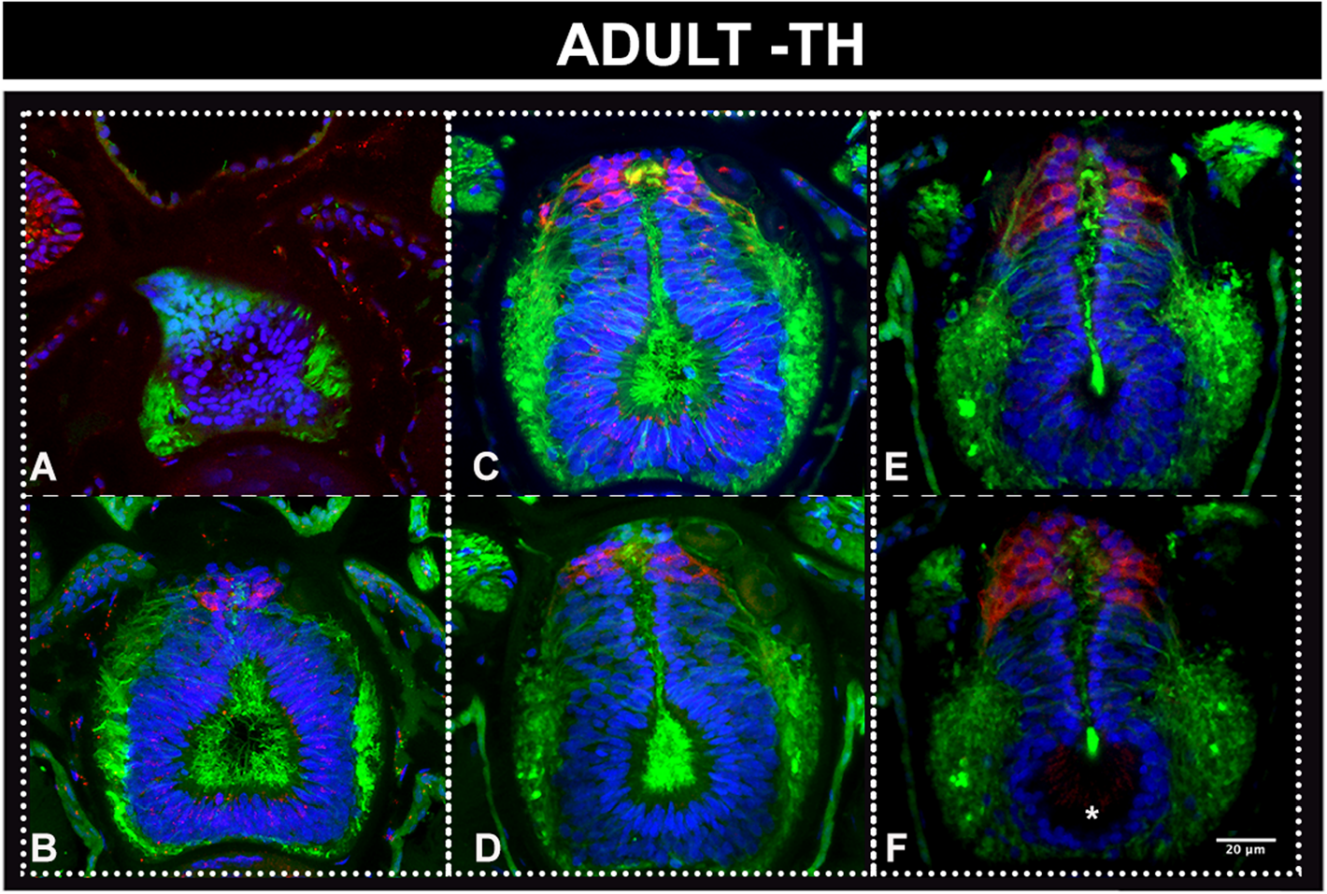
Dorsal accumulation of dopaminergic neurons in the adult amphioxus brain. **a**–**f** Neurons detected by immunohistochemistry with an antibody directed against tyroxine hydroxylase and shown in frontal thick vibratome sections ordered from anterior to posterior. The most anterior dopaminergic neurons are slightly posterior to the most anterior glutamatergic and GABAergic neurons, which are located at the level of the neuropore, as shown in Figs. 6d and 7h (sections at the same level as **a**). The dorsal domain of dopaminergic neurons starts at the level of **b**, equivalent to Figs. 6e and 7i for glutamatergic and GABAergic neurons, respectively, and broadens posteriorly with a more prominent dorsal cluster at the level of the infundibular organ, which is visible in **f** (asterisk). Colour code: red, TH; green, neuropil and intraventricular cilia (acetylated tubulin); blue/cyan, nuclear staining (Dapi)

Since our results suggest a sustained proliferation of neuronal progenitors, we investigated the degree of neuronal differentiation at different life cycle stages—with special emphasis on adults (Figs. 6, 7 and 8 and Additional files 8, 9 and 10 Figures S8-S10). We focused on the occurrence of key transmitters in terminally differentiated neurons, such as GABA, glutamate and acetylcholine, which we spatially related to a restricted population of dorsal dopaminergic neurons [58]. First, and consistent with a late development of specific domains in the amphioxus cerebral vesicle, we observed few differentiated neurons at early stages of development (as shown in one-gill-slit embryos in Figs. 6a–c, 7g and 8a, b). This is not surprising as these embryos are still developing. The differences in distribution are, however, striking when compared with those at metamorphic and adult stages, as is apparent for example in the case of glutamatergic and GABAergic neurons (Figs. 6 and 7).

In mice, 70–80% of cortical neurons (of telencephalic origin) are glutamatergic with their glutamatergic fate being set at their progenitor stage by the combinatorial action of *FoxG1*, *Lhx2*, *Pax6* and *Emx2* [59]. In amphioxus, only a small number of glutamatergic neurons are observed at embryonic stages: in the ventral-anterior floor of the cerebral vesicle, probably in association with the frontal eye (Fig. 6a–c), and ventrally in the neural tube, with paired clusters starting posteriorly to the post-infundibular part of the larval cerebral vesicle (Fig. 6a, b arrows). This agrees with previously published expression in earlier embryos [60]. In contrast, we observed several populations of glutamatergic neurons in the adult amphioxus brain and neural tube (Additional file 9 Figure S9). Most importantly, we found glutamatergic cells accumulating dorsally in the cerebral vesicle (Fig. 6d–i and arrows in Additional file 9 Figure S9), matching the area of overlap of *Lhx2*/9, *Pax4*/6 and *Emx* expression (see Additional file 10 Figure S10). Our data thus suggest that the cerebral vesicle in adult amphioxus has dorsal populations of glutamatergic neurons that may be molecularly specified similarly to those in vertebrate telencephalon. Posterior to the cerebral vesicle, we found small spindle-shaped spinal fluid contacting (CSF-) neurons (empty arrows in Additional file 9 Figure S9), bilaterally arranged glutamatergic neurons deeply embedded in the neuropile (arrows in Additional file 9 Figure S9) and, at the most dorsal level, scattered cells intermingling with the Joseph cells, probably corresponding to lamellate cells (LC in Additional file 9 Figure S9) (see for comparison description by [61]).

In addition, the mammalian cortex contains approximately 20% of GABAergic interneurons. These GABAergic interneurons have been shown to migrate from the MGE and LGE in several vertebrate species, including cyclostomes [62, 63]. In amphioxus, GABAergic neurons have been described at early stages of development, but these are not anteriorly located; instead, they have been found in the posterior end of the cerebral vesicle and in the neural tube [60, 64]. Our examination of GABAergic cells in one-gill-slit embryos confirmed a pair of GABAergic cells, bilaterally arranged (see inset in Fig. 7g), at the caudal end of the ventral cerebral vesicle (Fig. 7g). In adult brains, we observed instead a pronounced anterior and dorsal accumulation of GABAergic cells (Fig. 7a–o), complementing previous observations on the extensive GABAergic system in adult amphioxus [65]. We found GABAergic neurons occupying roughly the dorsal half of the anterior brain, with glutamatergic neurons partially overlapping in more dorsal areas (compare Fig. 6d–g with Fig. 7h–o), resembling the differential dorsoventral distribution observed in the mouse telencephalon [54].

Besides GABAergic cells, the vertebrate LGE also gives rise to dopaminergic interneurons that populate the olfactory bulb via the rostral migratory stream [29]. Dopaminergic cells have been previously identified in amphioxus via detection of tyrosine hydroxylase (TH), the rate-limiting enzyme of catecholamine biosynthesis, in strong correlation with dopamine immunoreactivity and very low noradrenaline content [58]. Again, the distribution of dopaminergic cells in the amphioxus CNS appears to significantly change throughout development and life cycle. In amphioxus larvae, dopaminergic cells populate a very small region posterior to the post-infundibular part of the larval cerebral vesicle [66], while in adults there are three different populations, the most anterior one spreading dorsally from the posterior half of the cerebral vesicle to a level in line with the junction between the first and the second myomere [58]. We set out to spatially relate this anterior dopaminergic population, described as population 1 by Moret and collaborators, to other neurons in the amphioxus cerebral vesicle, and found its restricted domain largely overlapping with that of the glutamate- and GABAergic cells (Fig. 8). Similar to the GABAergic neurons, the dopaminergic neurons were more roundish in shape as opposed to the columnar glutamatergic neurons (compare Fig. 8d to Fig. 6i). This opens up the possibility of cotransmission, as postulated for GABA and dopamine in the vertebrate olfactory bulb [67].

**Fig. 9:**
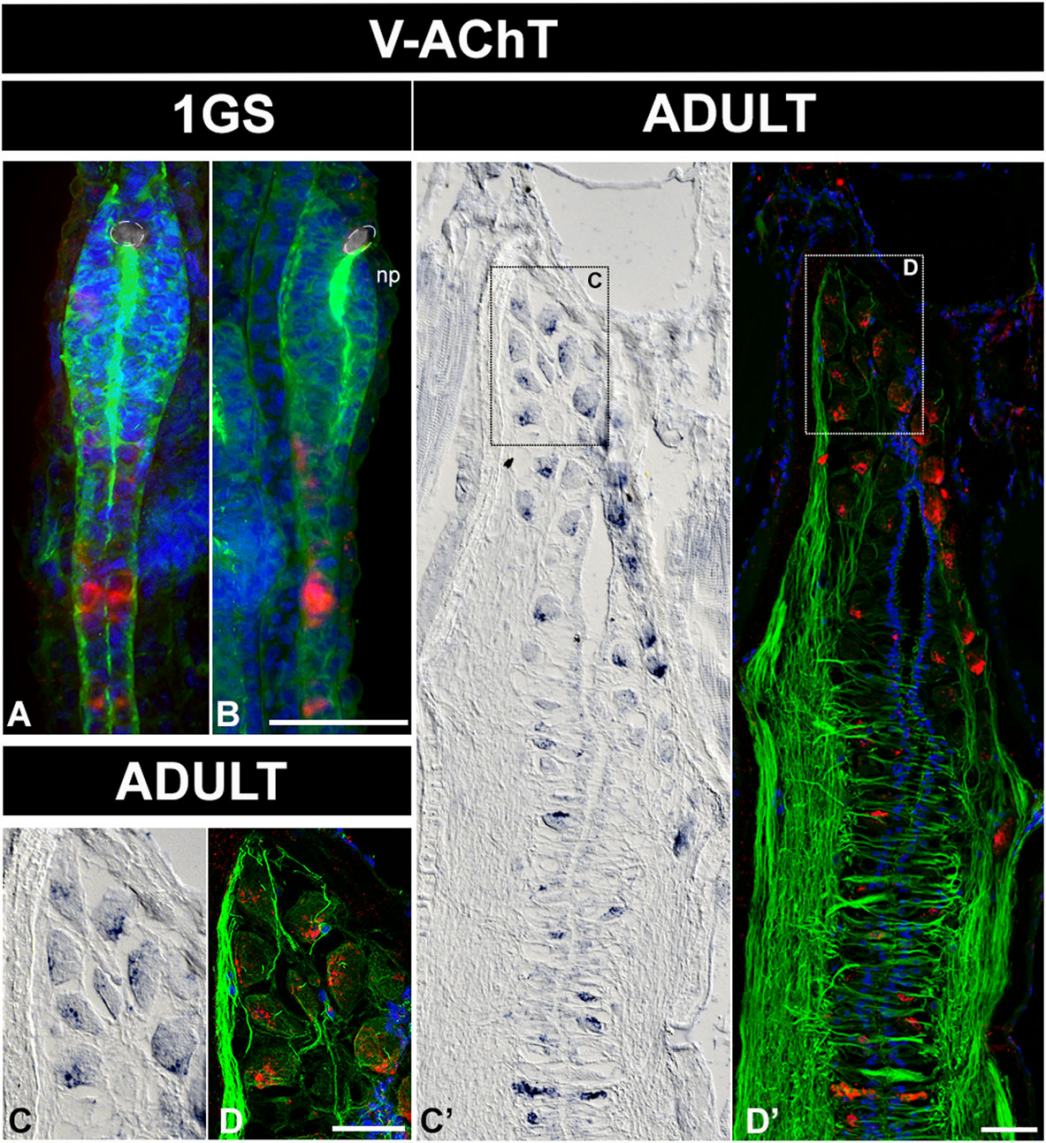
Cholinergic cells in the adult amphioxus brain accumulate dorsally in the Joseph cells mantle. **a, b** Distribution of cholinergic cells in one-gill-slit embryos (1GS) and in adult brains (**c**, **d**, **c’**, **d’**), detected by in situ hybridisation of *VAchT* in whole mount preparations and paraffin sections respectively. **a**, **b** In embryos, the only cholinergic cells detected are located in the neural tube. **c**, **d**, **c’**, **d’** In adult brains, we found no ventral cholinergic cells. All scale bars are 20μm. Colour code: red, VAchT; green, neuropil and intraventricular cilia (acetylated tubulin); blue/cyan, nuclear staining (Dapi). Abbreviation: np, neuropore

We detected another striking case of late differentiation with the cholinergic neurons, which are absent from the embryonic brain (Fig. 9a, b) but abundant in the brain roof of adults (Fig. 9c, d and c’, d’). These cells match the location and morphology described for the Joseph cells, which form the roof of the adult amphioxus brain and have been previously described as photoreceptors [26]. Notably, any relation between the Joseph cells and the cholinergic neurons of the vertebrate telencephalon (which are born ventrally to tangentially migrate to the striatum [54];) is at the moment unclear. Joseph cells use the same neurotransmitter, but they have a very particular morphology, and their relative position seems to differ from that of the cholinergic neurons in the vertebrate striatum. It is clear however that they develop late, as previously suggested [26], and most probably in association with the late developing dorsal part of the brain.

## Discussion

**Fig. 10:**
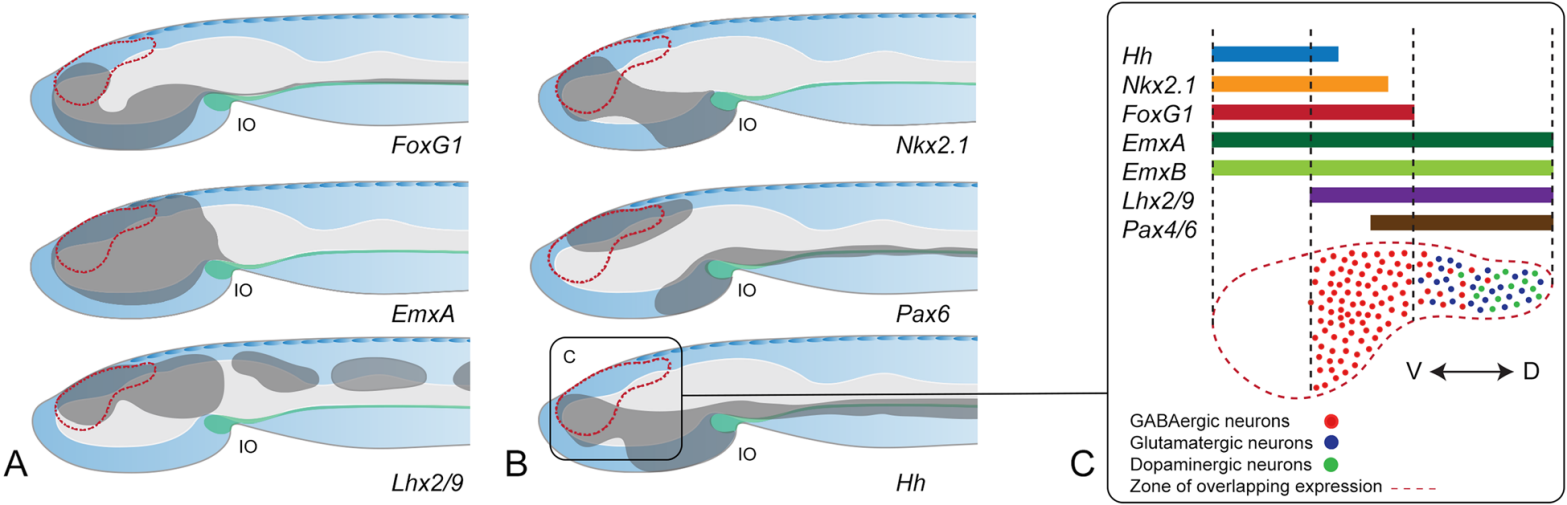
Combinatorial expression in the telencephalic-like region of the adult amphioxus brain. **A**, **B** Summary of expression patterns in Figs. 2 and 4 projected on a lateral schematic representation of an adult amphioxus brain (projected outline from the image in Fig. 5g). The grey shading represents the entire domain for each gene. The dashed red line indicates the region of expression overlap with a telencephalon-like profile. Joseph cells are shown in the most dorsal side of the brain, illustrating that they are positioned outside and dorsally to the telencephalon-like region (dashed red line). **C** Magnified view of the telencephalic-like region showing the dorsal-ventral distribution of gene expression and neurotransmitter identities. Abbreviation: IO infundibular organ

Our work identifies the PAD, a dorsoanterior part of the adult amphioxus cerebral vesicle that co-expresses a combination of conserved transcription factors that specify the telencephalon in vertebrates (*FoxG1*, *Emx*, *Lhx2/9*, *Fezf*) (Fig. 10). The PAD is regionalised by dorsal expression of *Pax6* and medio-ventral expression of *Nkx2.1*, transcription factors that in vertebrates segregate dorsal pallial versus ventral subpallial identities (Fig. 10). In addition, the ventral PAD also co-expresses the conserved *Hedgehog* ligand known to establish ventral identities in the vertebrate telencephalon (Fig. 10). Even if some of these genes also show sparse expression in embryonic brains, the whole network appears to only assemble through metamorphosis to be fully present at adult life cycle stages and only in this brain region.

Prior to this work, the expression of *FoxG1*, *Hh* and *Emx* was believed to be missing from the anterior amphioxus brain, contrasting with several reports of these genes being present in the anterior neuroectoderm in non-chordate invertebrates such as insects, annelids and enteropneusts [20, 23, 68]. This had fostered speculation that the amphioxus brain vesicle had lost expression of these factors secondarily [23]. Solving this conundrum, we now show that a similar expression profile to that of the vertebrate telencephalon is present in adult amphioxus.

### The amphioxus PAD—a possible homologue of the vertebrate telencephalon

Based on these data, we propose the homology of the *FoxG1, Emx, Lhx2/9—*expressing PAD in amphioxus to the telencephalon in vertebrates (Fig.  10). This hypothesis builds on the observation that the combined expression of these genes suggests a conserved role in the specification of this particular brain region in all chordates (with the exception of ascidians). Homology of the telencephalon and the PAD would imply that both structures descended from a ‘pre-telencephalic rudiment’ in the brain of the last common ancestor of the evolutionary lineages leading to vertebrates and cephalochordates.

What are the alternatives? First, the transcription factors may not have played an ancient role in tissue specification. Instead, they may have constituted an ancient system for axial patterning—as suggested for example for *Lhx2* and *Dach* that were proposed to belong to an ancient ‘anteriorposterior head patterning system’ [69]. In that case, these factors would have acted independent of later tissue identities. It is rather unlikely however that this applies to the transcription factor complement discussed here, given that in amphioxus the anteroposterior and dorsoventral axes are established at embryonic stages, long before *FoxG1* and *Emx* expression is initiated in the post-metamorphic brain. Instead, the late appearance of additional neuron populations concomitant with the late onset of transcription factor expression supports their role in tissue specification. Conditional gain and loss of function studies will be needed to settle this issue.

Second, the possibility has to be considered that the telencephalon and the PAD arose via convergent evolution, i.e. did not evolve from a common precursor structure. This would require the independent co-option, in the anterior brain of both amphioxus and vertebrates, of a pre-existing gene regulatory network from another developmental context, as suggested for example for the *Hox* genes in developing limbs [70]. Again, this notion appears to be unlikely in our case—first and foremost, because the combined expression of *FoxG1*, *Emx* and *Lhx2* is found no-where else in the body, neither in amphioxus, nor in the vertebrates, which means that there would have been no obvious context to co-opt it from. Instead, available data including evidence from annelid and enteropneust outgroups (see above) indicates that this particular network was already involved in the specification of particular neuronal types in the anterior neuroectoderm of ancestral chordates.

### Reconstructing the pre-telencephalic rudiment

The proposed homology would not imply any a priori structural or functional similarity of telencephalon and PAD, which had time to diverge for at least 500 million years after the separation of vertebrate and cephalochordate lineages. It would allow however for some (shared) features of the telencephalon and the PAD to be due to common descent rather than evolutionary convergence. What are these features? For reconstructing the pre-telencephalic rudiment, one has to consider data from all chordates. Tunicates however show an adult morphology that is an adaptation to their unique sessile lifestyle and have experienced multiple evolutionary losses including most of their adult brain, so comparisons to this group are not possible in this case. Thus, for the adult chordate brain, we are left with vertebrates and amphioxus to compare. Here, the reasoning is simple: Whatever trait we observe in both the amphioxus PAD and the vertebrate telencephalon is likely to come from a common ancestor and therefore a trait that was likely present in ancestral chordates.

Given that, what can be learned from the amphioxus-vertebrate comparison about ancestral characteristics of the adult pre-telencephalic rudiment? First, the amphioxus cerebral vesicle is organised anatomically similar to the vertebrate brain, as an enlarged anterior part of the neural tube. This means the pre-telencephalic rudiment represented the anterodorsal portion of the ancestral chordate neural tube. Second, histologically, the amphioxus PAD consists of periventricular cells surrounded by neuropil, like the pallium of lampreys, amphibians, some sharks and lungfishes [63]. Such anatomical organisation is particularly visible in adults, and acquired gradually throughout the life cycle, before and after metamorphosis, via sustained growth from the ventricular layer, which remains a mitotic niche and apparent source of neuronal progenitors. We thus hypothesise that a similar architecture might have already been present in the anterior brain of ancestral chordates. Third, just like telencephalon, the PAD is composed of glutamatergic, GABA- and dopaminergic neurons, indicating that this might have been also the case in the pre-telencephalic rudiment (Fig. 10). Fourth, as in vertebrates, glutamatergic neurons appear confined more dorsally whereas GABAergic neurons are found both dorsally and also more ventrally, where they overlap *Nkx2.1* expression (Fig. 10). This would suggest that the pre-telencephalic rudiment already showed a similar subdivision with territorially restricted neuronal types (which evolved into the pallial-subpallial subdivision in the vertebrate lineage). If so, it would be worth investigating a possible relation of the ventral PAD to the vertebrate basal ganglia, which are predominantly GABAergic and developmentally dependent on Nkx2.1 expression [71]. Fifth, in the amphioxus dorsal PAD, dopaminergic neurons co-localise with the GABA- and glutamatergic neurons. Notably, within the vertebrate pallium, this co-occurrence of the three transmitters is unique to the olfactory bulb. Adding to this, the periglomerular dopaminergic and GABAergic olfactory bulb cells (that derive from the rostral migratory stream) express *Dlx5/6* and *Emx1* in mouse—in line with the broad expression of *Emx* genes in the amphioxus PAD (our data) and locally restricted *Amphi-Dlx* expression in the dorsal cerebral vesicle of 2-gill-slit larvae [72]. Finally, the amphioxus PAD appears to be the target region of olfactory innervation, just like the olfactory bulb in vertebrates [73]. This would suggest that at least a large part of the dorsal part of the pre-telencephalic rudiment in chordate ancestors may have been olfactory bulb-like, with possible olfactory innervation.

### Telencephalon evolution via heterochrony?

Our data indicate a pronounced difference in developmental timing between vertebrate telencephalon and amphioxus PAD, with a late mode of specification observed in amphioxus as opposed to an early development of the telencephalic region in most vertebrates. In mice, for example, *Shh* is first expressed around E7.5 before inducing the expression of *FoxG1* in the overlying telencephalic anlage [38]. By E8.5, the telencephalic anlage, marked by the expression of *FoxG1*, is very large and is not yet divided into a set of paired vesicles [37]. One possibility is that this late development of the PAD in amphioxus is a specialisation of the amphioxus evolutionary lineage. Alternatively, a late specification may represent the ancestral chordate state. Interestingly, the expression of *FoxG1* has also been reported to occur relatively late in lampreys and in hagfish, reflecting a late specification of the telencephalon in relation to other vertebrates [74, 75]. Furthermore, in cartilaginous fish, teleosts and tetrapods, the onset of *foxG1* expression shows conspicuous variation [75]. In addition, most of the vertebrate telencephalic genes remain expressed at adult stages, with functions in neuronal survival and maintenance. *FoxG1*, for example, is expressed in the postnatal murine brain where it is involved in the survival of mature neurons and in the maintenance of GABAergic transmission [76]. *Emx1* and *Emx2* persist in the adult mouse cortex, with *Emx1* expanding further over time and *Emx2* remaining in the ventricular and marginal zones [77]. In adult medaka fish, *Lhx9* telencephalic expression persists in the mitral cells of the olfactory bulb [78]. Similarly, *Pax6* maintains the embryonic pattern of expression in adults, where it is involved in reparative neurogenesis and maintenance of mature neurons [79]. Altogether, the late expression of these genes appears to be a shared and ancestral feature of the chordate phylum.

## Conclusions

Our expression analysis of conserved genes that specify and regionalise telencephalon in the vertebrates opens up the exciting perspective that the PAD of the adult amphioxus cerebral vesicle represents the long-sought homologue of the vertebrate telencephalon in an invertebrate chordate. Shared features between the amphioxus PAD and the vertebrate telencephalon include the differential dorsoventral distribution of GABA- and glutamatergic neurons, and the co-occurrence of glutamate, GABA and dopamine transmitter types in a dorsal subpopulation of neurons that receives olfactory innervation.

In the future, it will be interesting to compare the neuronal circuitry of the amphioxus PAD with that of the vertebrate telencephalon, and to investigate how neurons in the amphioxus PAD connect to other parts of the amphioxus brain, including possible inhibitory GABAergic and/or dopaminergic subcircuits. Furthermore, it will be interesting to determine whether any form of neuronal migration occurs during PAD formation, for example towards the dorsal part of the brain vesicle—as observed during vertebrate telencephalic development.

## Materials and methods

### Collection, breeding, maintenance and staging of amphioxus

Wild catch collections of amphioxus were made in Banyuls-sur-mer (France), Helgoland (Germany) and Kristineberg (Sweden). Once transported to the laboratory, the adult amphioxus were maintained, bred and the progeny raised as described in [80] in a custom-made amphioxus facility.

This study used 208 adult amphioxus to reliably test for the expression patterns here published. The adults selected for the study were all sexually mature, between 2.5 and 3 cm long, and were sacrificed after spawning. For every other stage, at least 10–15 individuals were analysed in each case. All specimens were staged using morphological criteria (see Table 1) to avoid cofounding biases due to slight differences in the temperature of the incubators or due to parental fitness.

**Table 1:** Staging criteria for the specimens utilised in this study

Nomenclature	HPF @ 20C	Morphological criteria
8s	24h	8 somite embryos
1 GS	48h	Larvae with a well-formed first primary gill slit
2.5 GS	60h	Larvae with a well-formed first primary gill slit and the primordia of the second primary gill slit
5GS	3 months	Larvae with 5 primary gill slits
9-10 GS	6 months	Pre-metamorphic larvae with 10 primary gill slits**

*HPF* hours post-fertilisation

**B. lanceolatum larvae initiate metamorphosis when they have between 11 and 12 primary gill slits

### Gene cloning

Sequences of interest were identified in silico using a self-assembled transcriptome database generated in the laboratory [81]. Primers were designed accordingly and used to amplify the transcripts of interest from a custom-made SMARTer RACE (Clontech) cDNA library made from *B. lanceolatum* adult tissue. All genes were cloned in pCRII-TOPO TA vectors (Invitrogene, K4660-01) and in vitro transcribed using dual promoter sites for in situ probes. Probes were cleaned up using the RNeasy Kit (Quiagen, 74104).

### Adult tissue fixation

Previous to any fixations, all adults were anesthetised with MS-222 (A5040, Sigma) until irresponsive to a flash-light or touch. Heads were separated by decapitation and fixed either in 4%PFA-MOPS buffer, neutral buffered formalin (HT501128, Sigma), or 4%PFA-SeaWater for 24 h. We found formalin-fixed heads were more robust to subsequent embedding and processing, so all the images here reported are of heads fixed in formalin. Morphology was the same using either of the fixation methods. After fixation, adults were dehydrated through an ascending series of methanol and further dissected in 100% methanol. This last step of dissection was aimed at removing the ventral side of the head, by sectioning just underneath and along the notochord. This allowed to correct for any arching of the nervous system caused by the notochord shrinking during the fixation process. The sample could then be mounted flat on the ventral side with a corrected angle, so that the neural tube runs in parallel to the sectioning plane (see [35] for further details). Once the heads had been trimmed ventrally, these were kept in methanol until further processing.

### Embedding and paraffin sectioning

Dissected heads were prepared for embedding in paraplast by xylene series: xylene:ethanol (1:1), xylene, xylene:paraplast (1:1) and finally paraplast (P3558, Sigma). Thereafter, the prepared heads were transferred to a Leica Tissue Embedding Station (Leica EG1160) and mounted on their ventral side, as described above. Paraffin blocks were sectioned using a Leica rotatory microtome (RM255) at a thickness of 12–14μm. The sections were left to float in a 37 °C DEPC-treated water bath (Leica HI1210) and collected on SuperFrost Ultra Plus microscope slides (Menzel). The sections were flattened in a Leica flattening table (Leica HI1220) at 42 °C for approximately 1 h. Sections were further fixed to the slides overnight at 37 °C.

### Embedding and agarose sectioning

Dissected heads were prepared for embedding in 3% low melting agarose (A2790, Sigma), by rehydration through a descending series of methanol to 100% PBS. The heads were mounted, as described for paraffin preparations on their ventral side. Agarose blocks were sectioned in a Leica Vibratome (VT 1200S) at a thickness of 60–80μm. Floating sections were collected in gelatine-coated plates.

### In situ hybridisation

For whole mount in situ hybridisations, embryos were fixed and processed as in [82]. For in situ hybridisation on paraffin sections, paraffin was first dissolved by immersion in xylene. When sections looked clear, they were rehydrated through a descending series of ethanol to PBS. The tissue was pre-treated by immersion in HCl 0.2M for 15 min and later digested with 1μg/ml of Proteinase K at 37 °C for 40 min. The digestion was stopped by quick immersion in 0.2% glycine and glycine was thoroughly washed away with PBS. The subsequent hybridisation steps were followed as for the in situs in whole mount embryos.

### Immunohistochemistry

For immunohistochemistry in adult brains, vibratome floating sections were pre-treated by immersion in HCl, as explained above for in situs, and thoroughly washed afterwards in PBS-0.1%Triton-0.1%BSA. For both primary and secondary antibodies, the tissue was blocked in PBS-0.1%Triton-5%NGS and incubated overnight at 42 °C with the appropriate antibody. For whole mount immunohistochemistry, the brains were incubated for an extra overnight with each antibody. Antibodies were used as follows: anti-acetylated tubulin 1:250 (T 6793, Sigma), anti-PHH3 1:200 (ab5176, Abcam), anti-GABA 1:250 (ab8891, Abcam), anti-Glutamate 1:250 (G6642, Sigma), and anti-TH 1:150 (AB152, Chemicon). All secondary antibodies, goat anti-mouse-Alexa488 and goat anti-rabbit-Dylight549 (115-546-062 and 111-506-045, Jackson Immunoresearch), were used at 1:250.

### Edu labelling and detection

For Edu labelling, embryos were incubated in seawater containing Edu at a concentration of 10μM for 2 h before fixation. Embryos were harvested and fixed as for whole mount in situ hybridisation, in 4%PFA-MOPS Buffer. Fluorescent detection of the incorporated Edu was performed following the manufacturer instructions (Click-iT®EdU Alexa Fluor® 647 Imaging Kit, C10340, Invitrogen).

### Image acquisition and registration

Nomarski images were acquired with a Zeiss Imager M2 microscope. Confocal images were acquired with a Leica TCS SPE or a Leica TCS SP8. Light sheet images were acquired in a Z1-Zeiss microscope with a clarity module. For whole mount brain imaging in adults and pre-metamorphic embryos, tissues were clarified for a week in Focus Clear (Tebu-bio, F101-KIT). In all cases, sections, embryos and whole mounted brains were tile-scanned and the resulting hundreds of tiles per sample were assembled into single images either using the Leica LAS software, the Zeiss Zen software or the Stitching Plugins (3D and others) in ImageJ (Fiji) [83, 84].

## Supplementary Information

**Additional file 1: Figure S1.** Whole-head serial sectioning and staining for FoxG1. All coronal paraffin sections ordered from ventral to dorsal. The dark anterior pigment corresponds to the frontal eye (FE). Apart from the expression in the brain, FoxG1 is also expressed in some cells of the floor plate (FP) and some ventrolateral cells of the central canal. We also observed expression in somites (M), as described previously by Toresson et al., 1998. Abbreviations: CC: Central Canal; FP: Floor plate; FE: Frontal eye; IO: Infundibular organ; M: Muscle; N: Notochord; NP: Neuropore.

**Additional file 2: Figure S2.** Whole-head serial sectioning and staining for EmxA. All coronal paraffin sections ordered from ventral to dorsal. The dark anterior pigment corresponds to the frontal eye (FE). EmxA expression is very restricted to the anterior part of the brain only. The strongest staining is observed very dorsally, in sections 5 and 6. Abbreviations: FP: Floor plate; FE: Frontal eye; IO: Infundibular organ; M: Muscle; N: Notochord; NP: Neuropore.

**Additional file 3: Figure S3.** Whole-head serial sectioning and staining for EmxB. All coronal paraffin sections ordered from ventral to dorsal. The dark anterior pigment corresponds to the frontal eye (FE). EmxB expression is more restricted than EmxA, with signal only visible in sections 2 to 6. Abbreviations: FP: Floor plate; FE: Frontal eye; IO: Infundibular organ; M: Muscle; N: Notochord; NP: Neuropore.

**Additional file 4: Figure S4.** Whole-head serial sectioning and staining for Lhx2/9. All coronal paraffin sections ordered from ventral to dorsal. The dark anterior pigment corresponds to the frontal eye (FE). Apart from the expression in the brain, Lhx2/9 is also expressed in paired clusters of cells, resembling the pattern described for Lhx2 and Lhx9 in the zebrafish hindbrain (arrowheads). We also found Lhx2/9 left-right alternating clusters of cells, a pattern that follows the left-right offset of the somites (M) and nerve roots, therefore resembling the pattern of Lhx2 and Lhx9 reticulo-spinal neurons in the zebrafish neural tube (arrows). Abbreviations: FE: Frontal eye; HE: Hesse eyecups; IO: Infundibular organ; M: Muscle; N: Notochord; NP: Neuropore.

**Additional file 5: Figure S5.** Whole-head serial sectioning and staining for Pax6/4. All coronal paraffin sections ordered from ventral to dorsal. The dark anterior pigment corresponds to the frontal eye (FE). Apart from the expression in the brain, Pax4/6 is also expressed in some epidermal cells, probably sensory neurons, and very specifically in some lateral cells around the walls of the posterior ventricle. Abbreviations: CC: Central Canal; FP: Floor plate; FE: Frontal eye; HE: Hesse eyecups; IO: Infundibular organ; M: Muscle; N: Notochord; NP: Neuropore; PV: Posterior ventricle; SC: Epidermal sensory cells.

**Additional file 6: Figure S6.** Whole-head serial sectioning and staining for Nkx2.1. All coronal paraffin sections ordered from ventral to dorsal. The dark anterior pigment corresponds to the frontal eye (FE). Apart from the expression in the brain, Nkx2.1 might be also expressed in somites (M). Abbreviations: FE: Frontal eye; IO: Infundibular organ; M: Muscle; N: Notochord; NP: Neuropore.

**Additional file 7: Figure S7.** Whole-head serial sectioning and staining for Hh. All coronal paraffin sections ordered from ventral to dorsal. The dark anterior pigment corresponds to the frontal eye (FE). The pigments in the periventricular grey along the central canal (CC), posterior to the brain, correspond to the Hesse eyecups (HE). Hedgehog is also expressed in the floor plate (FP), posterior to the brain as previously described by Shimeld 1999. Abbreviations: CC: Central Canal; FP: Floor plate; FE: Frontal eye; HE: Hesse Eyecups; IO: Infundibular organ; M: Muscle; N: Notochord; NP: Neuropore.

**Additional file 8: Figure S8.** Whole-head serial sectioning and staining for VAchT. All coronal paraffin sections ordered from ventral to dorsal. The dark anterior pigment corresponds to the frontal eye (FE). The pigments in the periventricular grey along the central canal (CC), posterior to the brain, correspond to the Hesse eyecups (HE). No expression was detected in the ventral side of the cerebral vesicle and most of the dorsal cholinergic cells observed are Joseph cells. VAChT is also expressed by specialised (uncharacterised) columnar cells located in the periventricular grey of the central canal. Abbreviations: CC: Central Canal; FP: Floor plate; FE: Frontal eye; HE: Hesse Eyecups; IO: Infundibular organ; JC: Jospeh Cells; M: Muscle; N: Notochord; NP: Neuropore.

**Additional file 9: Figure S9.** Whole-head serial sectioning and staining for VGluT. All coronal paraffin sections ordered from ventral to dorsal. Within the brain most VGluT positive cells are located in the most anterior dorsal part of the vesicle (arrowheads). Outside from the cerebral vesicle glutamatergic cells are found at different levels: in a ventral to dorsal direction some small rounded glutamatergic cells are visible in the neuropil (arrows) and in the ventricular zone; a bit more dorsally there are also spindle-shaped spinal fluid contacting (CSF-) neurons (empty arrows); and at the roof of the brain in lamellate cells (LC) that intermingle with VGluT negative Joseph cells. Abbreviations: CC: Central Canal; FP: Floor plate; FE: Frontal eye; HE: Hesse Eyecups; IO: Infundibular organ; JC: Joseph Cells; LC: Lamellate Cells; M: Muscle; N: Notochord; NP: Neuropore.

**Additional file 10: Figure S10.** Full series of serially sectioned brains for FoxG1, EmxA, EmxB, Lhx2/9, Pax4/6, Nkx2.1, Hh, Fezf, VGluT and VaChT. All brains are shown in serial coronal paraffin sections from ventral to dorsal, with the ventricle of the cerebral vesicle (cv) centred in the images. In all cases the anterior part of the brain is at the top of the image. The top diagrams are a schematic representation of the morphology of the sections in a ventral to dorsal direction. Next to them it is the picture of an adult amphioxus head showing the plane of sectioning of the brain, which is highlighted in yellow, as explained in Figure 1. For clarity only sections where expression was detected are shown, accordingly the VAChT series starts only at dorsal levels. The scale bar for amphioxus sections is 50μm.

## Data Availability

Sequences are deposited in Ensemble Metazoan under the following IDs: BL11067 (FoxG1), BL18410 (Lhx2/9), BL15126 (Nkx2.1), BL13922 (Pax4/6), BL90339 (V-GluT), and BL06963 (Hh); or in the GeneBank under the following accession numbers: MW574992 (EmxA), MW574993 (EmxB), and MW574994 (VAchT). Images are deposited in the image repository Dryad: 10.5061/dryad.t1g1jwt1j.

## Abbreviations

CC: Central canal
CNS: Central nervous system
CSP: Contacting spinal fluid
FE: Frontal eye
FP: Floor plate
HE: Hesse eyecups
IO: Infundibular organ
JC: Joseph cells
LC: Lamelate cells
LGE: Lateral ganglionic eminence
M: Muscle
MGE: Medial ganglionic eminence
N: Notochord
NP: Neuropore
PAD: Pars anterodorsalis of the amphioxus brain
PV: Posterior ventricle
SC: Epidermal sensory cells
TH: Tyrosine hydroxylase
