# Supplementary figures and images for: The dorsoanterior brain of adult amphioxus shares similarities in expression profile and neuronal composition with the vertebrate telencephalon
Source: BMC Biol. 2021 May 21;19:110. doi: 10.1186/s12915-021-01045-w (PMC8139002; doi:10.1186/s12915-021-01045-w)

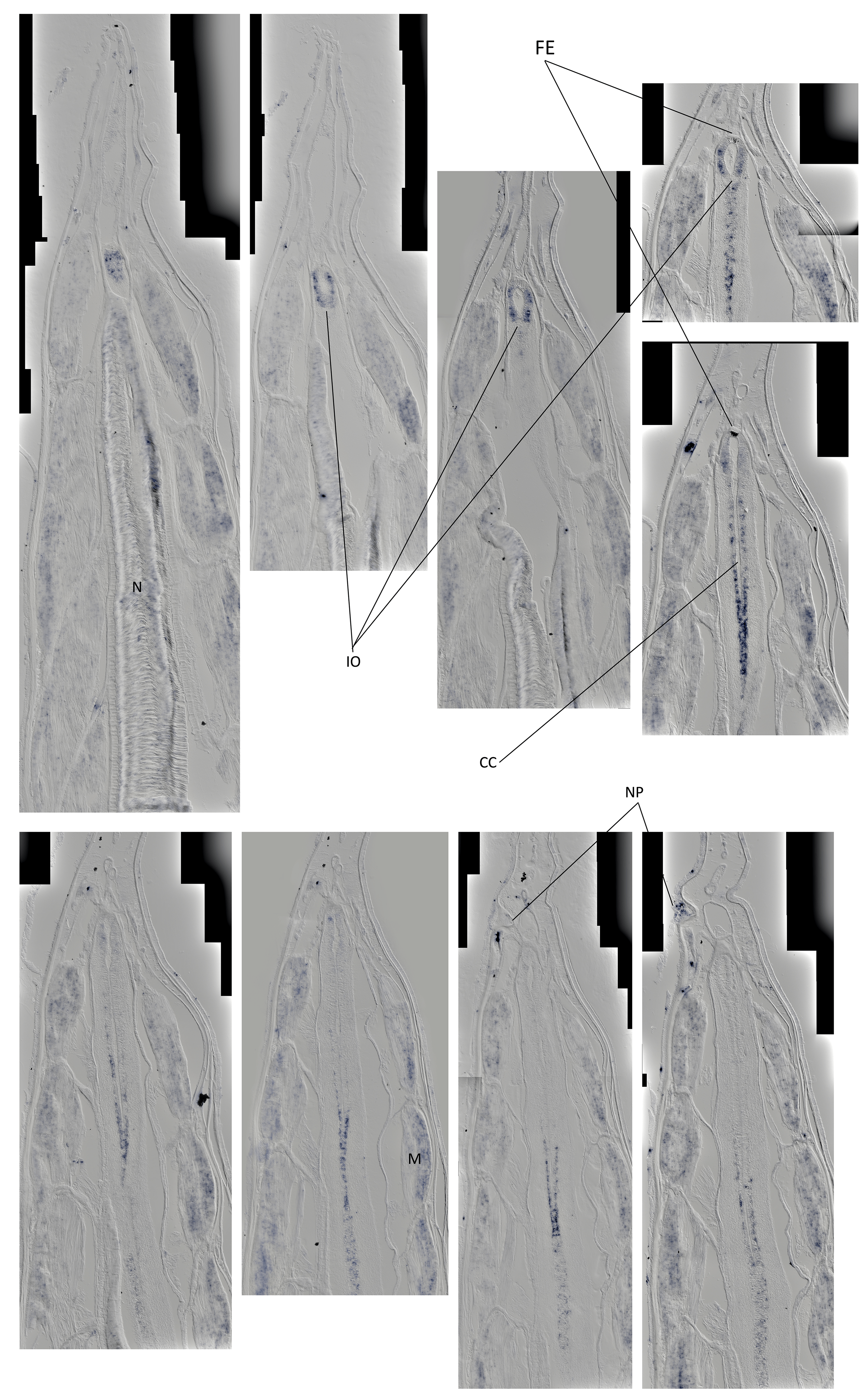

Supplement: Supplementary file 1 — Additional file 1: Figure S1. Whole-head serial sectioning and staining for FoxG1. All coronal paraffin sections ordered from ventral to dorsal. The dark anterior pigment corresponds to the frontal eye (FE). Apart from the expression in the brain, FoxG1 is also expressed in some cells of the floor plate (FP) and some ventrolateral cells of the central canal. We also observed expression in somites (M), as described previously by Toresson et al., 1998. Abbreviations: CC: Central Canal; FP: Floor plate; FE: Frontal eye; IO: Infundibular organ; M: Muscle; N: Notochord; NP: Neuropore. [file 12915_2021_1045_MOESM1_ESM.png]

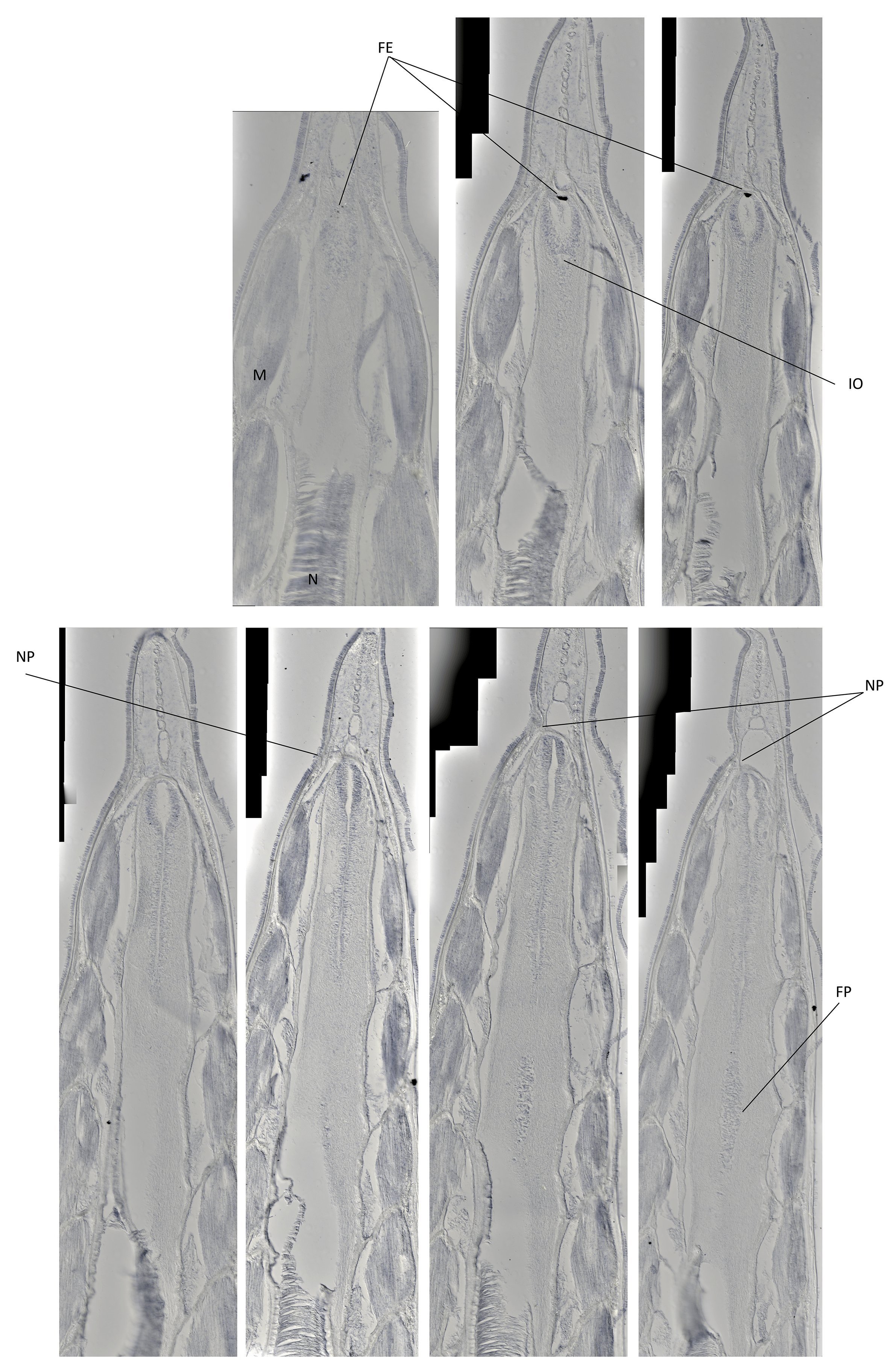

Supplement: Supplementary file 2 — Additional file 2: Figure S2. Whole-head serial sectioning and staining for EmxA. All coronal paraffin sections ordered from ventral to dorsal. The dark anterior pigment corresponds to the frontal eye (FE). EmxA expression is very restricted to the anterior part of the brain only. The strongest staining is observed very dorsally, in sections 5 and 6. Abbreviations: FP: Floor plate; FE: Frontal eye; IO: Infundibular organ; M: Muscle; N: Notochord; NP: Neuropore. [file 12915_2021_1045_MOESM2_ESM.png]

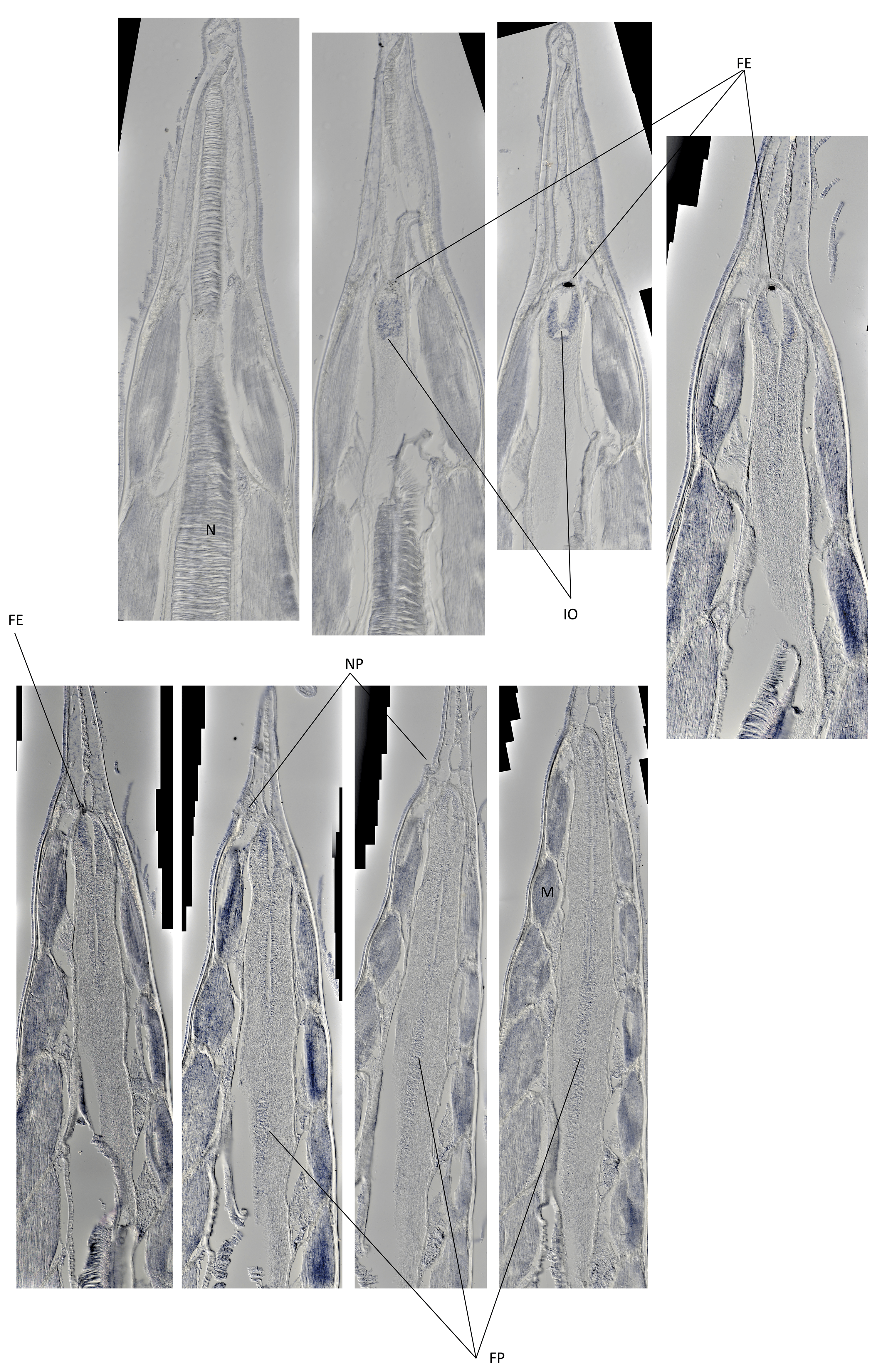

Supplement: Supplementary file 3 — Additional file 3: Figure S3. Whole-head serial sectioning and staining for EmxB. All coronal paraffin sections ordered from ventral to dorsal. The dark anterior pigment corresponds to the frontal eye (FE). EmxB expression is more restricted than EmxA, with signal only visible in sections 2 to 6. Abbreviations: FP: Floor plate; FE: Frontal eye; IO: Infundibular organ; M: Muscle; N: Notochord; NP: Neuropore. [file 12915_2021_1045_MOESM3_ESM.png]

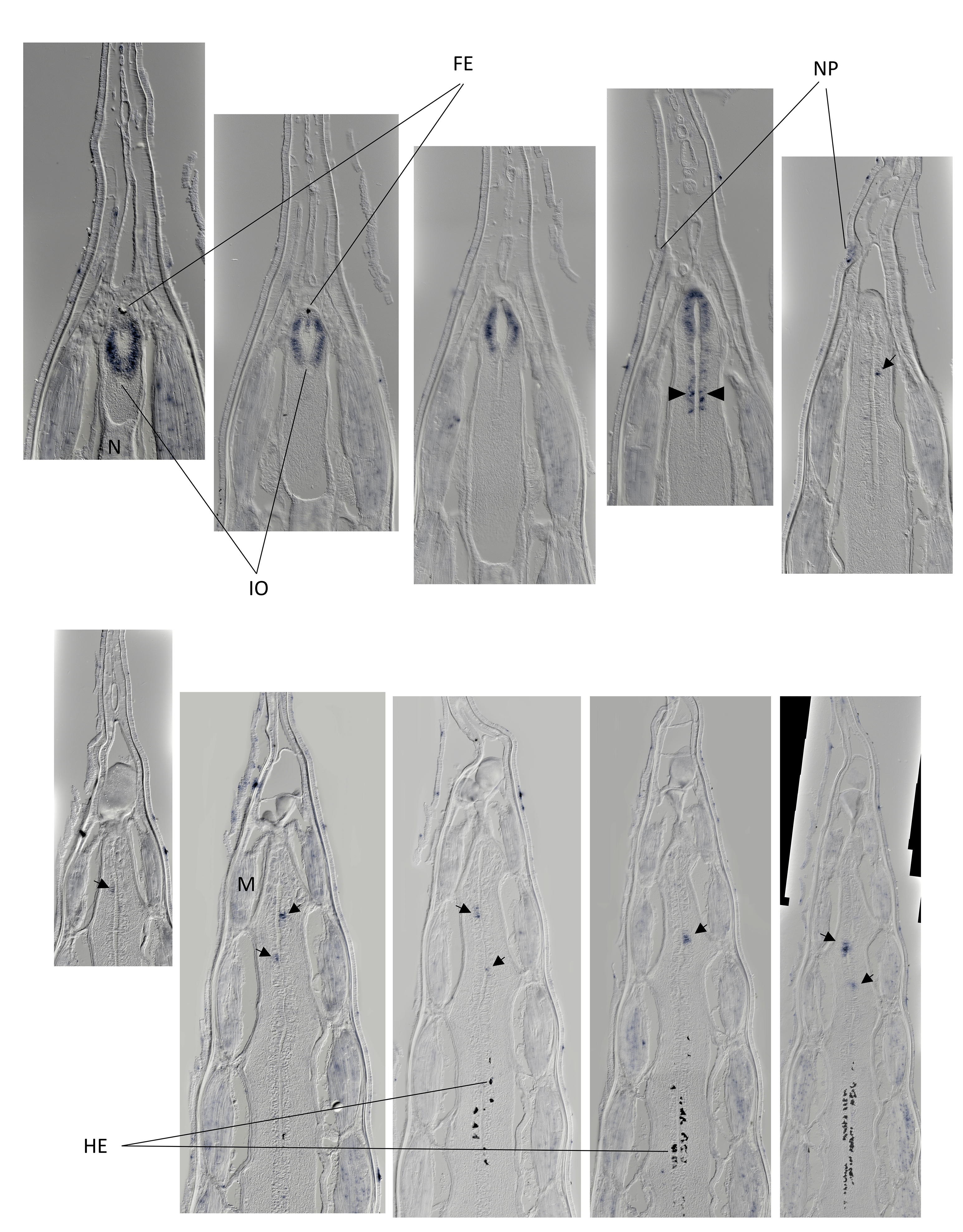

Supplement: Supplementary file 4 — Additional file 4: Figure S4. Whole-head serial sectioning and staining for Lhx2/9. All coronal paraffin sections ordered from ventral to dorsal. The dark anterior pigment corresponds to the frontal eye (FE). Apart from the expression in the brain, Lhx2/9 is also expressed in paired clusters of cells, resembling the pattern described for Lhx2 and Lhx9 in the zebrafish hindbrain (arrowheads). We also found Lhx2/9 left-right alternating clusters of cells, a pattern that follows the left-right offset of the somites (M) and nerve roots, therefore resembling the pattern of Lhx2 and Lhx9 reticulo-spinal neurons in the zebrafish neural tube (arrows). Abbreviations: FE: Frontal eye; HE: Hesse eyecups; IO: Infundibular organ; M: Muscle; N: Notochord; NP: Neuropore. [file 12915_2021_1045_MOESM4_ESM.png]

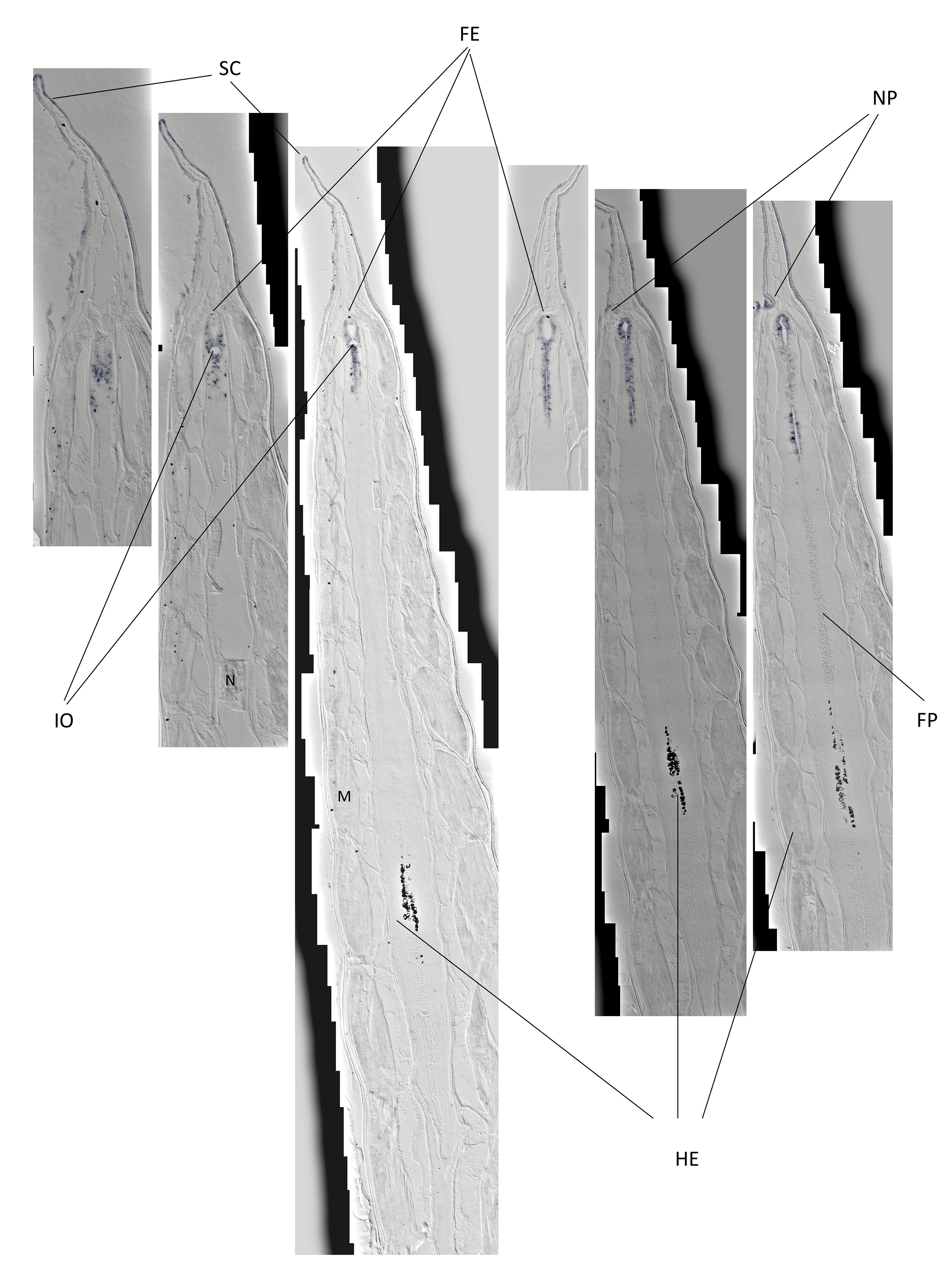

Supplement: Supplementary file 5 — Additional file 5: Figure S5. Whole-head serial sectioning and staining for Pax6/4. All coronal paraffin sections ordered from ventral to dorsal. The dark anterior pigment corresponds to the frontal eye (FE). Apart from the expression in the brain, Pax4/6 is also expressed in some epidermal cells, probably sensory neurons, and very specifically in some lateral cells around the walls of the posterior ventricle. Abbreviations: CC: Central Canal; FP: Floor plate; FE: Frontal eye; HE: Hesse eyecups; IO: Infundibular organ; M: Muscle; N: Notochord; NP: Neuropore; PV: Posterior ventricle; SC: Epidermal sensory cells. [file 12915_2021_1045_MOESM5_ESM.png]

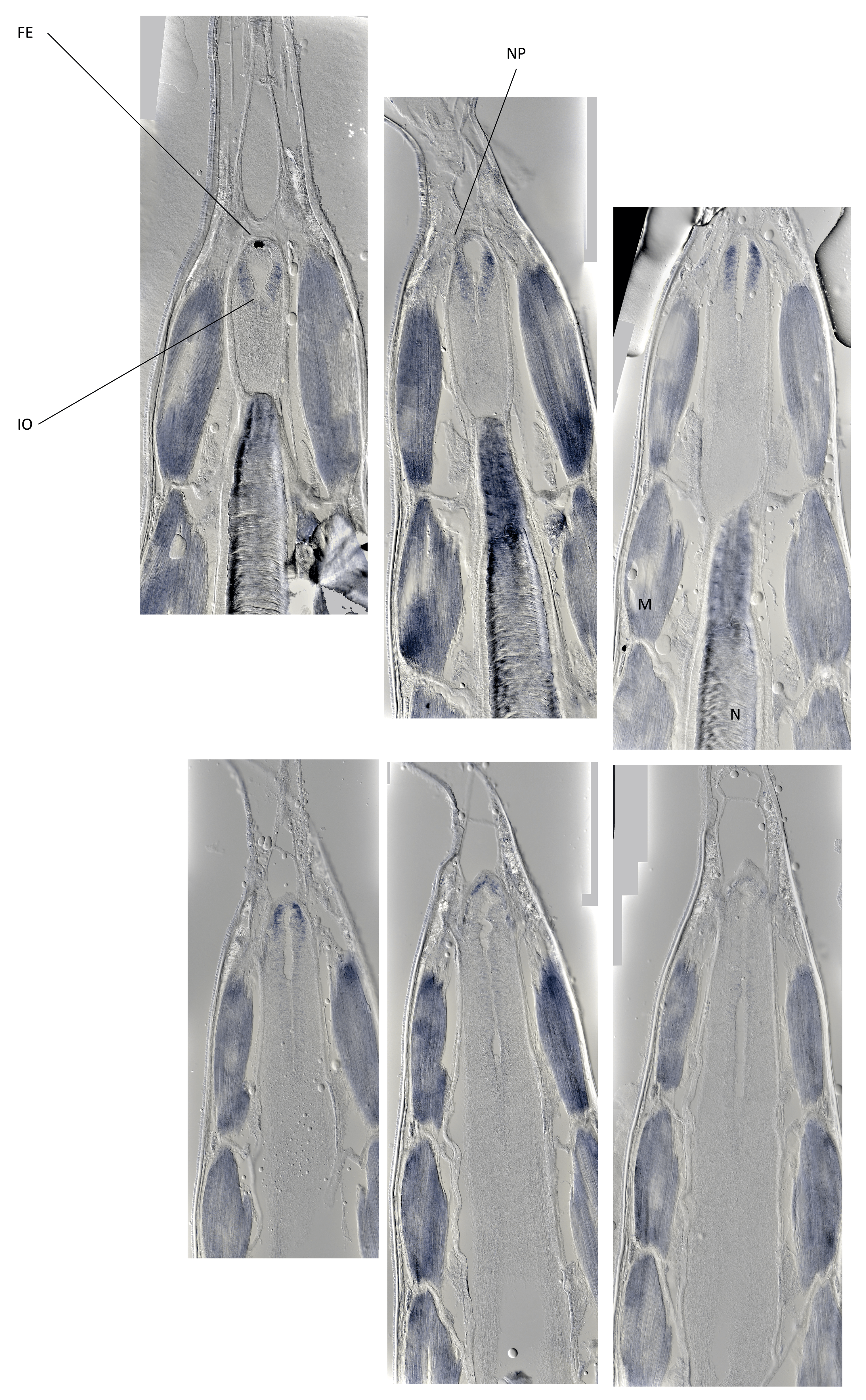

Supplement: Supplementary file 6 — Additional file 6: Figure S6. Whole-head serial sectioning and staining for Nkx2.1. All coronal paraffin sections ordered from ventral to dorsal. The dark anterior pigment corresponds to the frontal eye (FE). Apart from the expression in the brain, Nkx2.1 might be also expressed in somites (M). Abbreviations: FE: Frontal eye; IO: Infundibular organ; M: Muscle; N: Notochord; NP: Neuropore. [file 12915_2021_1045_MOESM6_ESM.png]

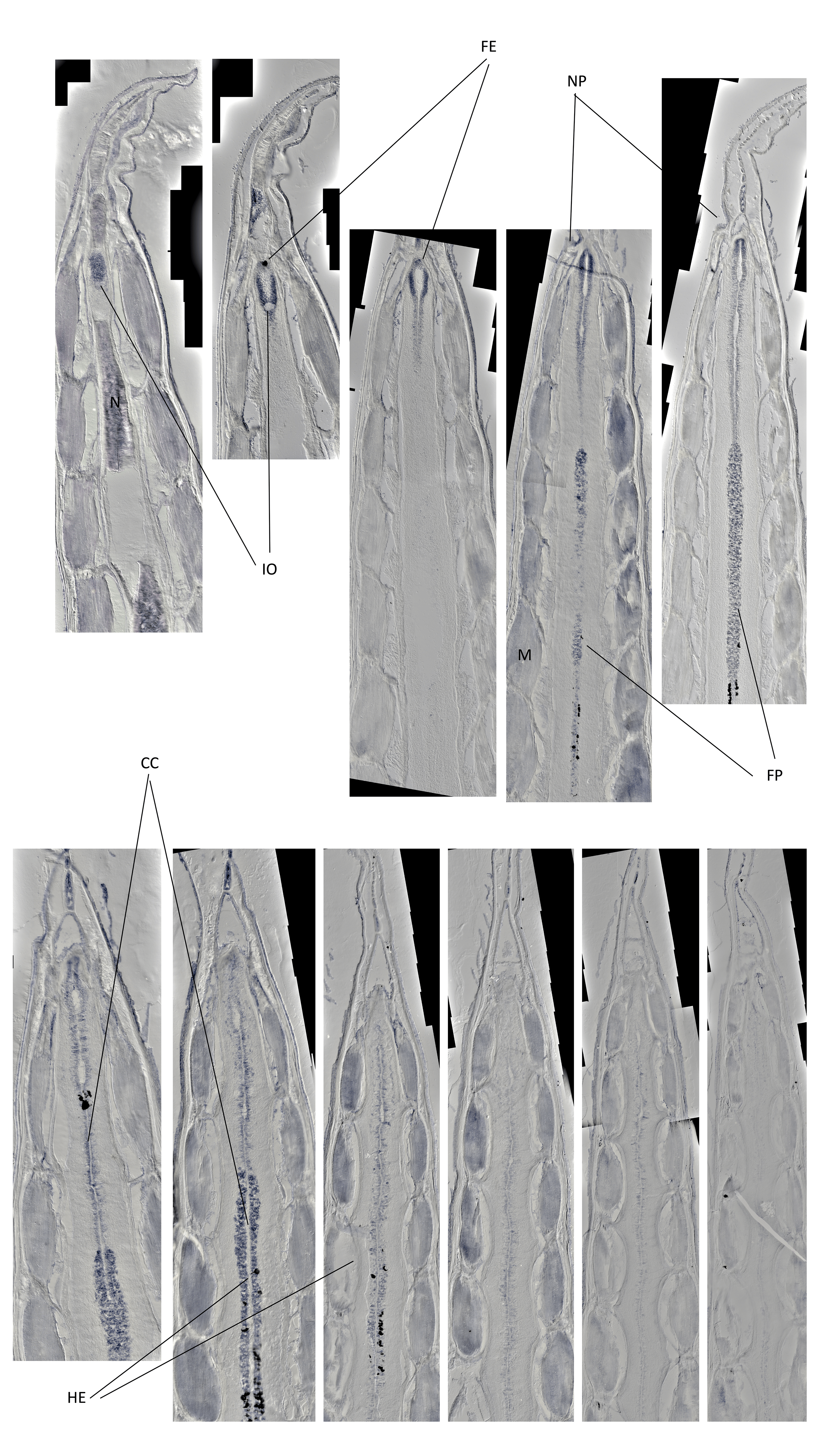

Supplement: Supplementary file 7 — Additional file 7: Figure S7. Whole-head serial sectioning and staining for Hh. All coronal paraffin sections ordered from ventral to dorsal. The dark anterior pigment corresponds to the frontal eye (FE). The pigments in the periventricular grey along the central canal (CC), posterior to the brain, correspond to the Hesse eyecups (HE). Hedgehog is also expressed in the floor plate (FP), posterior to the brain as previously described by Shimeld 1999. Abbreviations: CC: Central Canal; FP: Floor plate; FE: Frontal eye; HE: Hesse Eyecups; IO: Infundibular organ; M: Muscle; N: Notochord; NP: Neuropore. [file 12915_2021_1045_MOESM7_ESM.png]

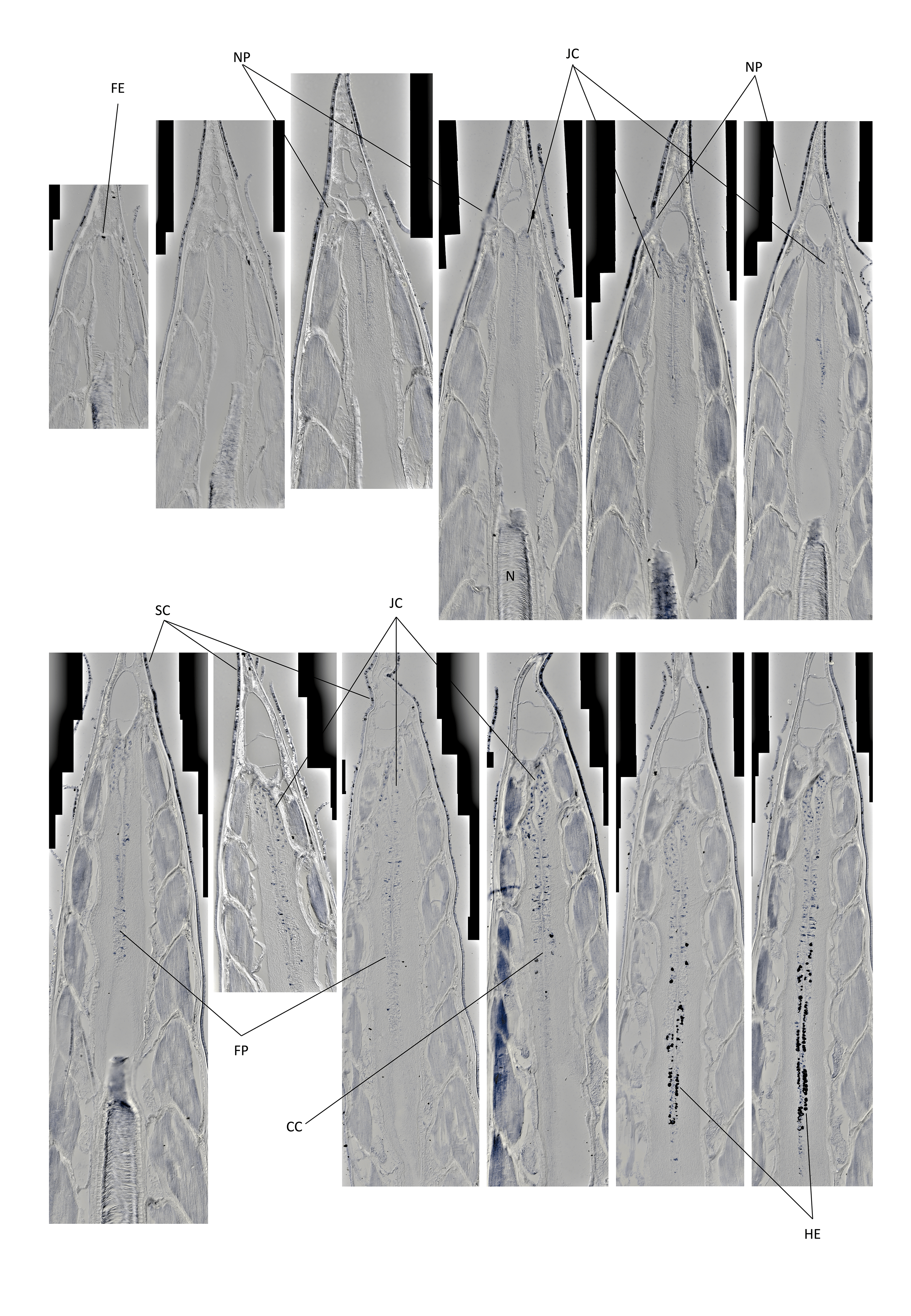

Supplement: Supplementary file 8 — Additional file 8: Figure S8. Whole-head serial sectioning and staining for VAchT. All coronal paraffin sections ordered from ventral to dorsal. The dark anterior pigment corresponds to the frontal eye (FE). The pigments in the periventricular grey along the central canal (CC), posterior to the brain, correspond to the Hesse eyecups (HE). No expression was detected in the ventral side of the cerebral vesicle and most of the dorsal cholinergic cells observed are Joseph cells. VAChT is also expressed by specialised (uncharacterised) columnar cells located in the periventricular grey of the central canal. Abbreviations: CC: Central Canal; FP: Floor plate; FE: Frontal eye; HE: Hesse Eyecups; IO: Infundibular organ; JC: Jospeh Cells; M: Muscle; N: Notochord; NP: Neuropore. [file 12915_2021_1045_MOESM8_ESM.png]

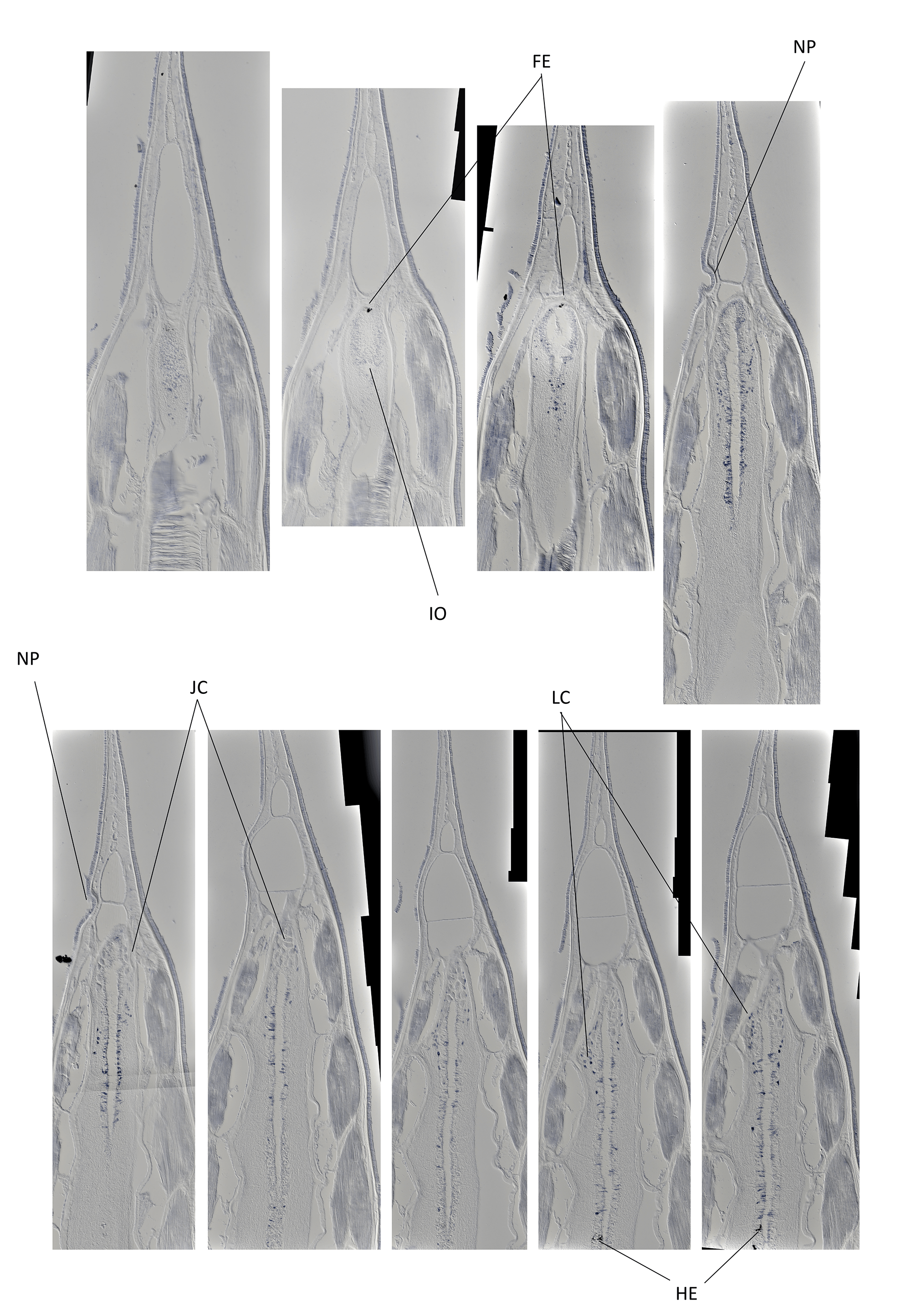

Supplement: Supplementary file 9 — Additional file 9: Figure S9. Whole-head serial sectioning and staining for VGluT. All coronal paraffin sections ordered from ventral to dorsal. Within the brain most VGluT positive cells are located in the most anterior dorsal part of the vesicle (arrowheads). Outside from the cerebral vesicle glutamatergic cells are found at different levels: in a ventral to dorsal direction some small rounded glutamatergic cells are visible in the neuropil (arrows) and in the ventricular zone; a bit more dorsally there are also spindle-shaped spinal fluid contacting (CSF-) neurons (empty arrows); and at the roof of the brain in lamellate cells (LC) that intermingle with VGluT negative Joseph cells. Abbreviations: CC: Central Canal; FP: Floor plate; FE: Frontal eye; HE: Hesse Eyecups; IO: Infundibular organ; JC: Joseph Cells; LC: Lamellate Cells; M: Muscle; N: Notochord; NP: Neuropore. [file 12915_2021_1045_MOESM9_ESM.png]

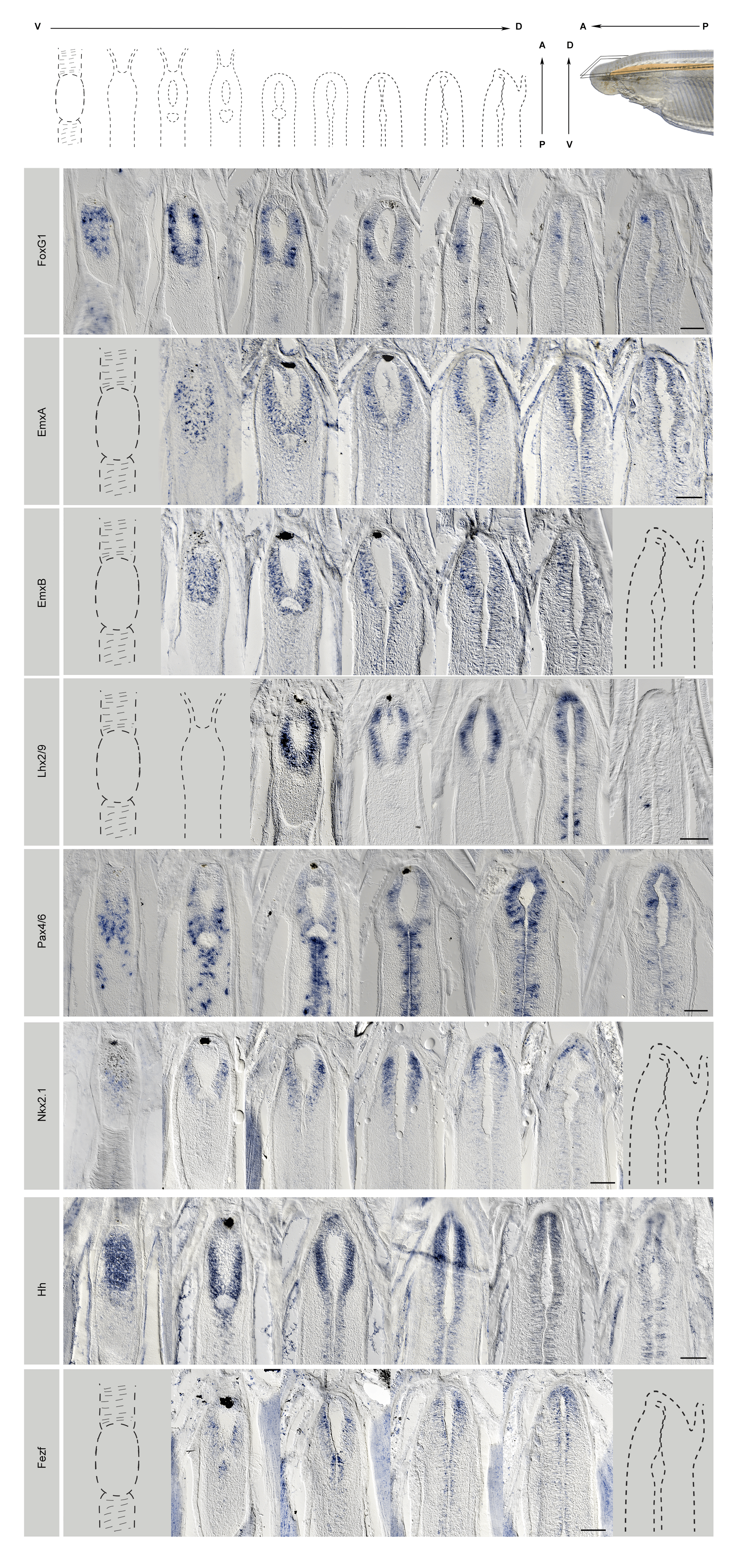

Supplement: Supplementary file 10 — Additional file 10: Figure S10. Full series of serially sectioned brains for FoxG1, EmxA, EmxB, Lhx2/9, Pax4/6, Nkx2.1, Hh, Fezf, VGluT and VaChT. All brains are shown in serial coronal paraffin sections from ventral to dorsal, with the ventricle of the cerebral vesicle (cv) centred in the images. In all cases the anterior part of the brain is at the top of the image. The top diagrams are a schematic representation of the morphology of the sections in a ventral to dorsal direction. Next to them it is the picture of an adult amphioxus head showing the plane of sectioning of the brain, which is highlighted in yellow, as explained in Figure 1. For clarity only sections where expression was detected are shown, accordingly the VAChT series starts only at dorsal levels. The scale bar for amphioxus sections is 50μm. [file 12915_2021_1045_MOESM10_ESM.png]
